# d-Alanine content in the marine edible bivalve *Panopea japonica* and evaluation of its associated enzyme activities

**DOI:** 10.1038/s41598-025-10379-2

**Published:** 2025-07-14

**Authors:** Mayu Onozato, Tomoya Takaura, Wataru Shinohara, Takehiro Tsukada, Tatsuya Sakamoto, Kenji Okoshi, Takeshi Fukushima

**Affiliations:** 1 Department of Analytical Chemistry, Faculty of Pharmaceutical Sciences, Toho University, 2-2-1 Miyama, Funabashi-shi, 274-8510 Chiba Japan; 2 Chiba Municipal Chiba High School, 9-46-1 Konakadai, Inage-ku, Chiba-shi, 263-0043 Chiba Japan; 3 Department of Biomolecular Science, Faculty of Science, Toho University, 2-2-1 Miyama, Funabashi-shi, 274-8510 Chiba Japan; 4 Department of Environmental Science, Faculty of Science, Toho University, 2-2-1 Miyama, Funabashi-shi, 274-8510 Chiba Japan; 5 Toyo Institute of Food Technology, 4-23-2 Minami-Hanayashiki, Kawanishi-shi, 666-0026 Hyogo Japan

**Keywords:** d-Alanine, Bivalve, Amino acid, Siphon, Alanine racemase, d-amino acid oxidase, LC-MS/MS, Marine biology, Bioanalytical chemistry

## Abstract

Amino acids play essential roles in various biological processes. In humans, most amino acids are present in the l-form; however, small amounts of d-amino acids also exist and have significant physiological roles, highlighting the importance of dietary intake from foods or drinks. In this study, we investigated the amino acid composition of the geoduck clam *Panopea japonica*, emphasizing its remarkably high d-alanine (d-Ala) content in the siphon tissue. The d-Ala content (6.99–14.2 mmol/100 g-wet) amounted to 91–94% of the total Ala, far exceeding that of other bivalves such as *Tresus keenae* (74%). Enzyme assays revealed alanine racemase and d-amino acid oxidase activities, suggesting active d-Ala biosynthesis and metabolism. The high concentrations of d-Ala enhance its value as a delicacy owing to its unique sweetness. This study provides new insights into the biosynthesis and metabolic characteristics of d-Ala in bivalves, highlighting its physiological and food ingredient significance.

**Supplementary Information:**

The online version contains supplementary material available at 10.1038/s41598-025-10379-2.

## Introduction

Amino acids are low molecular weight compounds that play various important roles in nutrition, neurotransmission, and immunity. Furthermore, they are the constituents of proteins. In humans, most amino acids are present in the l-form. However, a small amount of d-amino acids also exist, playing crucial physiological roles. For example, it has been reported that in schizophrenia, a major psychiatric disease, the serum concentration of d-serine (d-Ser), an endogenous co-agonist for *N*-methyl-d-aspartate (NMDA) receptors, is decreased^1,2^. Furthermore, the co-administration of antipsychotic drugs with d-Ser or d-alanine (d-Ala) has been shown to improve clinical symptoms^3,4^. Our previous study revealed that serum d-Ser levels are already decreased in individuals with an at-risk mental state (ARMS)^5^, a phase suggested as a transition stage toward psychosis/schizophrenia, although it does not yet meet the formal diagnostic criteria. Such findings highlight the significant impact of d-amino acids on human health and disease treatment^6^. Based on these reports, it is considered that a daily intake of d-amino acids from food and other sources may affect the onset of psychiatric diseases. In addition to their physiological relevance, d-amino acids are found in various products, including fermented foods such as vinegar, lactic acid bacteria beverage, *miso*, cheese, and black garlic^7,8^. Among these d-amino acids, d-Ala is an essential osmolyte in bivalves^9^ and crustaceans^10^, both of which are edible seafoods, and has been frequently detected in aquatic organisms. However, little is known about its specific distribution and function across the different tissues and species.

**Fig. 1:**
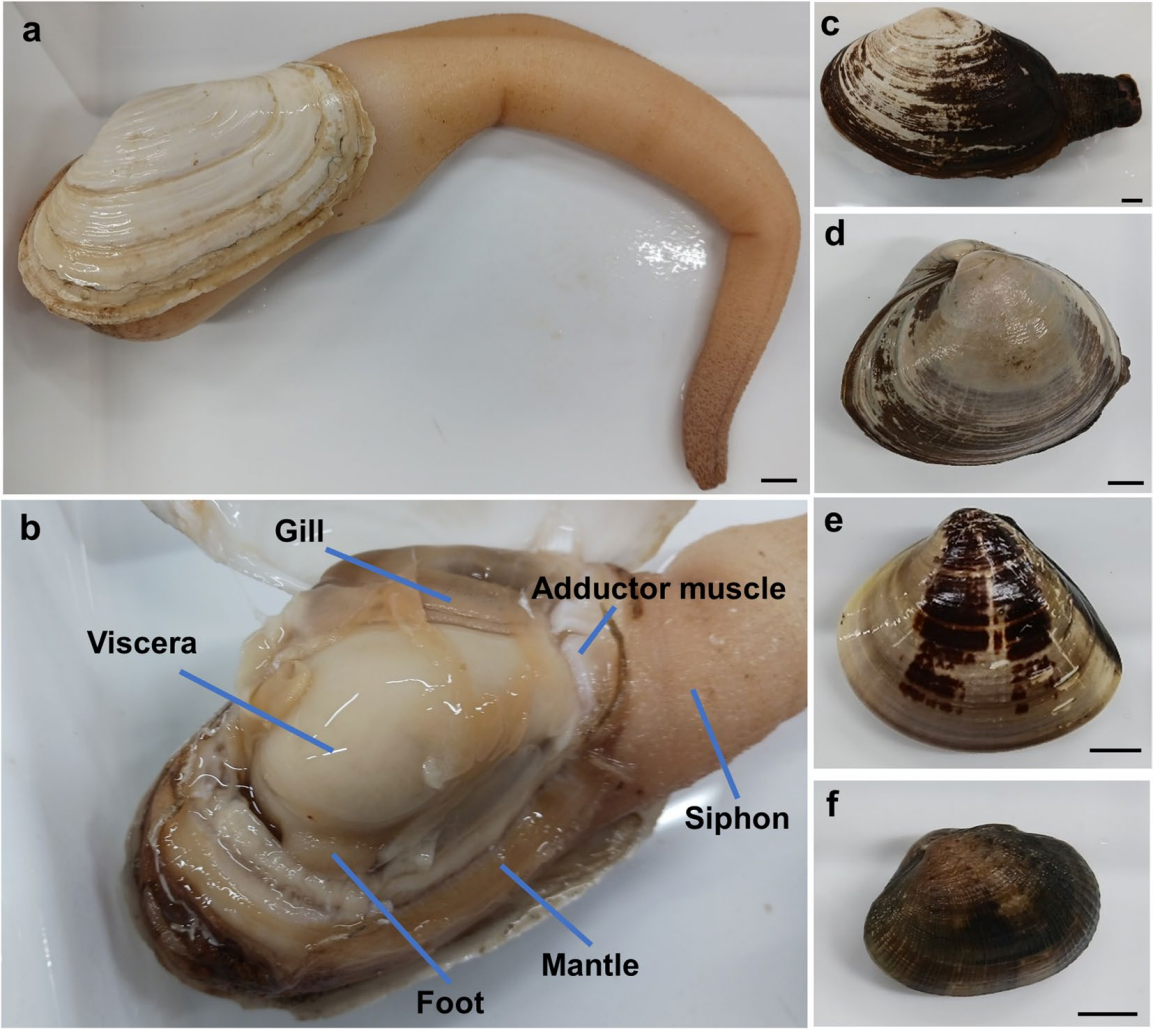
Digital images of the different species included in this study. (**a**) *Panopea japonica* immediately after removal from sea water. (**b**) *P. japonica* with the shell opened (the six parts sampled are shown). The siphon was sampled 1 cm closer to the shell from the tip. (**c**) *Tresus keenae*, (**d**) *Spisula sachalinensis*, (**e**) *Meretrix lusoria*, and (**f**) *Ruditapes philippinarum.* Bars indicate 1 cm.

*Panopea japonica*, a geoduck clam (Fig. 1a), is an infaunal species inhabiting in subtidal zones along the coasts of Japan, northern China, Korea, and Russia, particularly in sandy and muddy sediments at depths of 10 to 50 m^11^. Adults, with a shell length of 13–16 cm and a weight of 300–800 g^12^, possess a long siphon that enables filter feeding in deep sediments^13^. This siphon has attracted attention as a culinary delicacy, traditionally prepared as sashimi or lightly boiled for sushi, owing to its rich amino acid content and unique texture^13^. However, few studies have investigated the distribution, concentration, and physiological functions of d-amino acids in *P. japonica*, leaving a gap in our understanding of their biological and nutritional significance.

In this study, we investigated the amino acid composition of various tissues in *P. japonica*, comparing it with other bivalves to characterize its unique features. In particular, we focused on the high concentration of d-Ala in its siphon tissue and examined the activities of alanine racemase (AR) and d-amino acid oxidase (DAO) involved in its biosynthesis and degradation. By integrating biochemical and ecological perspectives, this study provides new insights into the physiological adaptations and commercial value of *P. japonica*.

## Methods

### Chemicals and reagents

The derivatization reagent^14^ and fluorescence probe^15^ used in the evaluation of the DAO activity were synthesized in our laboratory as previously reported. l-Alanine^13^C,*d*_1_ (l-Ala^13^C,*d*_1_) was purchased from MedChem Express (Monmouth Junction, NJ, USA). Guaranteed reagent-grade hydrochloric acid (HCl) was obtained from FUJIFILM Wako Pure Chemical (Osaka, Japan). Trizma^®^ base, l-glutathione reduced, and bovine serum albumin (BSA) were procured from Sigma-Aldrich (St. Louis, MO, USA). Flavin adenine dinucleotide (FAD) was purchased from Nakalai Tesque, Inc. (Kyoto, Japan). The details for the reagents used for the determination of amino acids in biological samples and those not listed above are provided in the Supplementary Information.

### Sampling and homogenate preparation of bivalves and Kuruma prawn

*Panopea japonica* samples from Aichi Prefecture (Japan) were purchased from a local fish shop in Taito-ku, Tokyo or online in April, June, and August 2024 (Fig. 1a, b). *Tresus keenae* samples from Aichi Prefecture were purchased from the same local fish shop in April 2024 (Fig. 1c). In May 2024, *Spisula sachalinensis* (Fig. 1d) were collected from the beach around Nagahama and *Meretrix lusoria* (Fig. 1e) were collected from the tidal flats of the Mangoku-ura Lagoon in Miyagi Prefecture. *Ruditapes philippinarum* samples were also collected from Mangoku-ura Lagoon in Miyagi Prefecture in May (Fig. 1f) and Chiba Port, Tokyo Bay, in June 2024 by us. Furthermore, *R. philippinarum* samples from Nemuro (Hokkaido) were also purchased from the Tsukiji market, Tokyo in June 2024. All bivalves were refrigerated at 4℃ until dissection. Their shell dimensions and whole wet weight were measured (Supplementary Table S1). A spatula-like shell peeler was used to open the shells, and the siphon, foot, adductor muscle, mantle, gill, and viscera (each ~ 200 mg) were collected for preparing the homogenate. In July 2024, kuruma prawns (*Marsupenaeus (Penaeus) japonicus*) from Oita Prefecture were purchased at Tsukiji market. Their total wet weight was measured, and hepatopancreas and muscle samples (each ~ 200 mg) were collected. The homogenate was prepared as previously reported^16^ and the supernatant was diluted five-fold with PBS and stored at − 80 ℃ until analysis.

### Determination of amino acids

Following previously reported methods, the thawed samples (10 µL) were deproteinized, derivatized with (*R*)-CIMa-OSu, and their amino acid contents were determined using liquid chromatography tandem mass spectrometry (LC-MS/MS)^8^. The concentrations of amino acids in each species of bivalve and kuruma prawn were calculated as the mean of the concentrations (mmol/100 g-wet) of the independent subjects (mean ± SD, *n* = 2 ~ 3).

### Cluster analysis of amino acid concentrations in various tissues of bivalves

Cluster analysis was performed for each tissue based on the amino acid concentrations of 20 bivalve individuals. The Bray–Curtis similarity index^17^, the significance of the differences between groups in the cluster^18^, and the components that contributed to the separation of groups were analyzed following the methods described in our previous report^16^.

### Assay of AR activity

The AR activity was determined using the method described by Nomura et al.^19^ with some modifications. Briefly, 50 mM tris-HCl buffer (pH 8.0, 480 µL) containing 10 mM l-Ala^13^C,*d*_1_ (10 µL), and either 10 µL of diluted *P. japonica* viscera or PBS were mixed in 2.0-mL tubes. After incubation at room temperature for 0, 1, 3, 6, 9, and 15 h (two independent *P. japonica* individuals, *n* = 3 each duration), the samples were boiled at 95 ℃ for 5 min, centrifuged (13,200 × *g*, 4 ℃, 15 min), and the supernatant was stored at − 80 ℃. The same procedure was followed for the siphon of *P. japonica* and the hepatopancreas and muscle of kuruma prawn for 0 and 15 h. The methods for measuring d-Ala^13^C,*d*_1_ by derivatization with CIMa-OSu^8,14^ are described in the Supplementary Information. The protein content of the homogenate was measured using the Bradford method with BSA as the standard, according to the manufacturer’s instructions (TaKaRa Bradford Protein Assay Kit, Takara Bio, Shiga, Japan). The peak area of d-Ala^13^C,*d*_1_, which increased with the addition of the homogenate, was then divided by the protein content to calculate the AR activity.

### Assay of d-amino acid oxidase activity

The DAO activity was determined using MeS-d-KYN^15^, based on the conversion of d-KYN to KYNA following a modified method from our previous report^20^. The DAO fraction was prepared following our previously reported method with minor modifications^21^, described in the Supplementary Information. Briefly, 100 mM tris-HCl buffer (pH 8.3, 260 µL), 10 mM FAD in H_2_O (10 µL), 1.0 mM GSH in H_2_O (10 µL), 20 mg/mL BSA in H_2_O (10 µL), and the prepared DAO sample (200 µL) from bivalves and kuruma prawn were mixed (*n* = 4). After incubation at 37 ℃ for 15 min, 10 mM MeS-d-KYN in DMSO (10 µL) was added and incubated at 37 ℃ for 60 min. H_2_O (1.0 mL) was added, and the mixture was boiled at 95 ℃ for 5 min to stop the reaction. The solution was centrifuged (2,500 × *g*, 4 ℃, 5 min) and the supernatant was diluted four times with mobile phase A (20 mM ammonium formate in H_2_O/MeOH (90/10, *v*/*v*)) and filtrated. MeS-KYNA was determined using an HPLC-fluorescence detector (the detailed analytical conditions are described in the Supplementary Information) following our previously reported method with minor modifications^22^. The protein content of the DAO fraction was measured as described above, and the DAO activity was also corrected for the protein content.

## Results

### *P. japonica* structure

Figure 1a shows a picture of the purchased *P. japonica* placed on a tray after being removed from seawater. When immersed in seawater, *P. japonica* retained seawater inside its siphon and shell, with the volume corresponding to approximately half of its soft-body wet weight^23^. The siphon was very thick and extended to a length approximately two to three times the shell length when filled with sea water, however, it became shrunken and rigid after draining the water. Upon carefully cutting through the adductor muscle to open the shell, the thin membrane was removed and a milky-white, ball-shaped viscera was found in the center (Fig. 1b). An extremely small foot was attached to the end of the viscera (Fig. 1b). Two gills were present on each side and a thick siphon was connected to the mantle margin (Fig. 1b). The mantle was very thick and it was impossible to close the shell completely.

### Free amino acid concentration and ratio in tissues of bivalves and Kuruma prawn

**Fig. 2:**
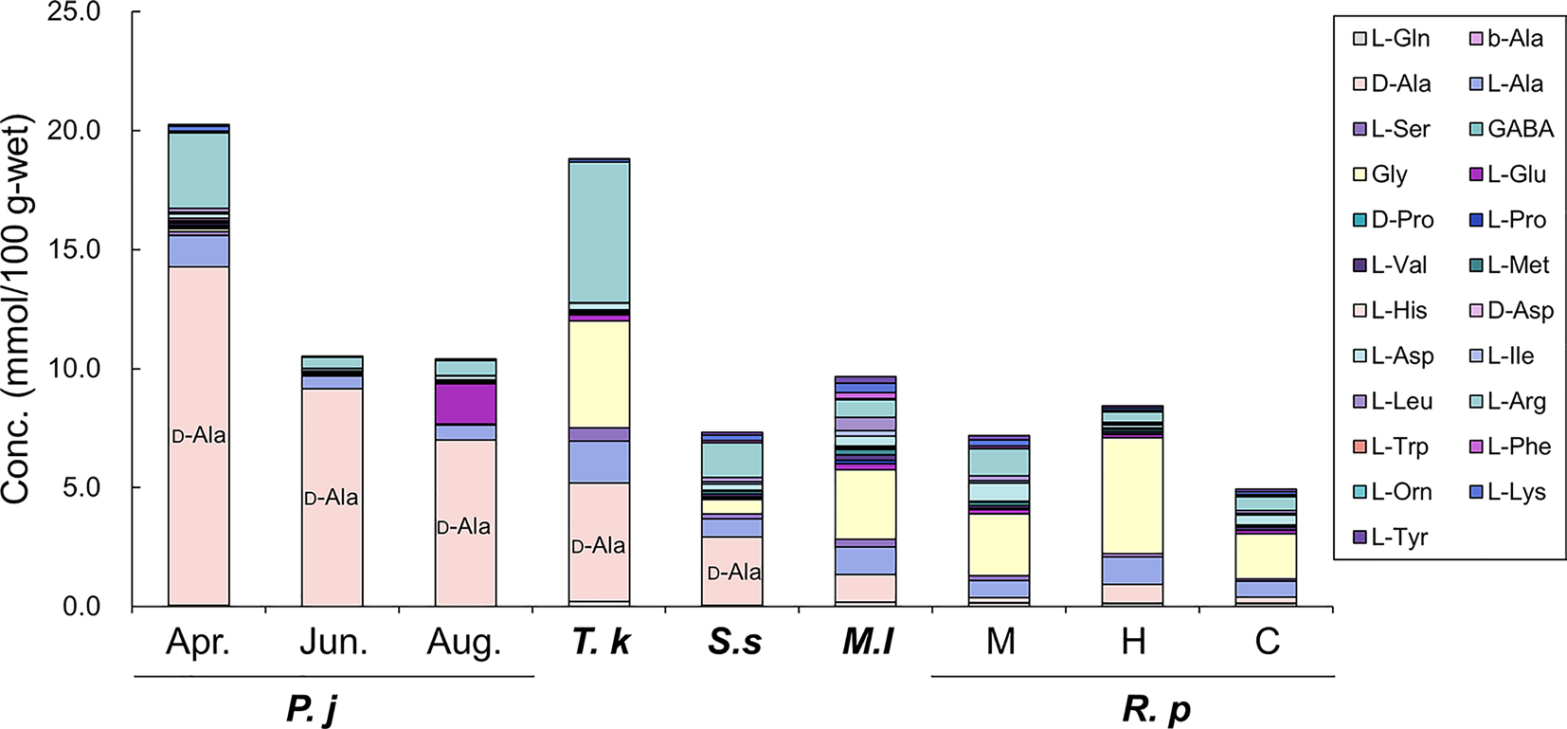
The amino acids detected in the siphon of bivalves (mmol/100 g-wet). ***P. j***: *Panopea japonica*; ***T. k***: *Tresus keenae*; ***S. s***: *Spisula sachalinensis*; ***M. l***: *Meretrix lusoria*; ***R. p***: *Ruditapes philippinarum*. The M, H, and C on ***R. p*** indicate their habitat, i.e. Miyagi, Hokkaido, and Chiba prefectural areas, respectively.

The amino acid concentrations in the various tissues of bivalves are presented in Supplementary Tables S2a–f. The concentration of amino acids in each bivalve tissue is shown in Fig. 2 (siphon) and Supplementary Figs. S2a–S2e (foot, adductor muscle, mantle, gill, and viscera, respectively), and the percentage of each amino acid to the total concentration is shown in Supplementary Figs. S3a–S3f. The total concentration of amino acids, namely a mixture of the l- and d-forms, in the siphon of *P. japonica*, *T. keenae*, *S. sachalinensis*, *M. lusoria*, and *R. philippinarum* was 10–20, 19, 7, 10, and 5–8 mmol/100 g-wet, respectively (Fig. 2), while the percentage of d-Ala in each siphon was 67–87, 26, 39, 12, and 3–9%, respectively (Supplementary Fig. S3a).

**Fig. 3:**
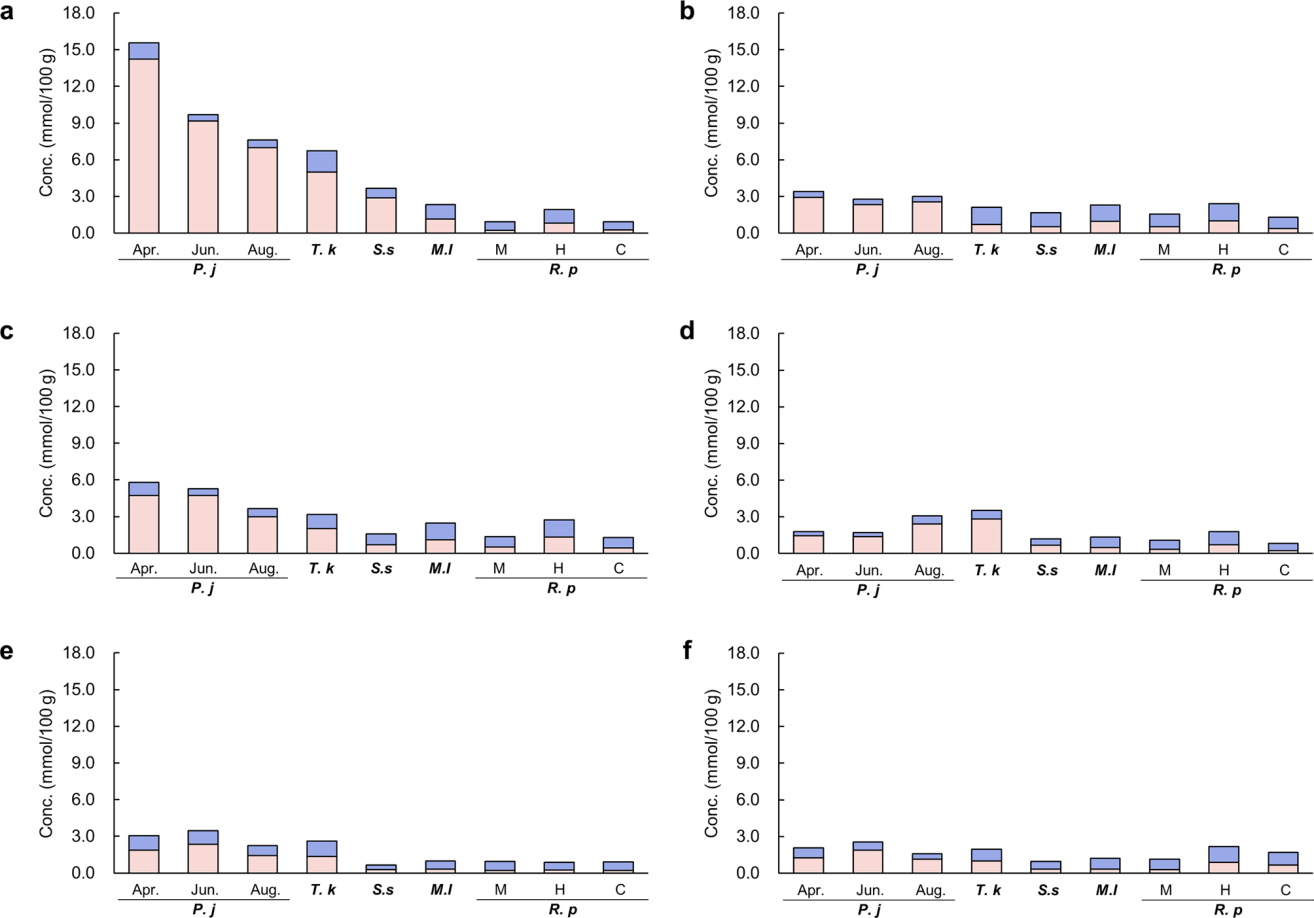
d-Ala and l-Ala concentrations detected in the various tissues of bivalves (mmol/100 g-wet). The abbreviations refer to Fig. 2. (**a**) siphon, (**b**) foot, (**c**) adductor muscle, (**d**) mantle, (**e**) gill, and (**f**) viscera.

**Fig. 4:**
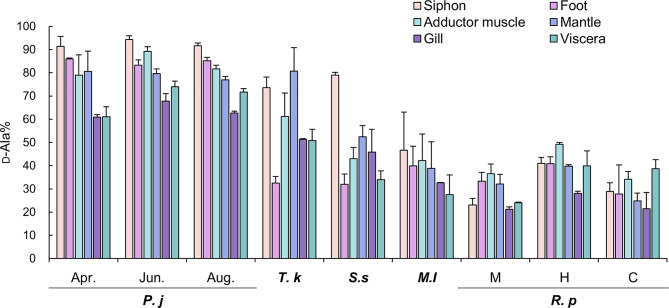
The percentage of d-Ala to total Ala (d-Ala + l-Ala) detected in each tissue of bivalves. The abbreviations refer to Fig. 2.

The concentrations of d-Ala and l-Ala detected in each of the six tissues are shown in Fig. 3. The concentrations of d-Ala in the siphon, foot, adductor muscle, mantle, gill, and viscera of *P. japonica* were 6.99–14.2, 2.32–2.92, 2.97–4.74, 1.38–2.42, 1.42–2.35, and 1.15–1.87 mmol/100 g-wet, respectively. The concentrations of l-Ala in the siphon, foot, adductor muscle, mantle, gill, and viscera of *P. japonica* were 0.53–1.33, 0.44–0.48, 0.57–1.07, 0.32–0.68, 0.84–1.11, and 0.45–0.81 mmol/100 g-wet, respectively. The percentage of d-Ala to total Ala (d-Ala + l-Ala) (d-Ala%) in each tissue of each bivalve is shown in Fig. 4. d-Ala% in the siphon of *P. japonica*, *T. keenae*, *S. sachalinensis*, *M. lusoria*, and *R. philippinarum* was 91–94, 74, 79, 47, and 23–41%, respectively. The concentrations of d- and l-Pro and d- and l-Asp detected in each of the six tissues are shown in Supplementary Figs. S4 and S5, and the d-Pro% and d-Asp% are presented in Supplementary Fig. S6.

The amino acid concentrations in the muscle and hepatopancreas of the kuruma prawn samples are presented in Supplementary Table S3. The concentrations of d-Ala in the muscle and hepatopancreas were 0.50 ± 0.12 and 0.30 ± 0.14 mmol/100 g-wet, respectively, while d-Ala% was 45 ± 2.6 and 14 ± 0.79, respectively.

### Cluster and SIMPER analyses

**Fig. 5:**
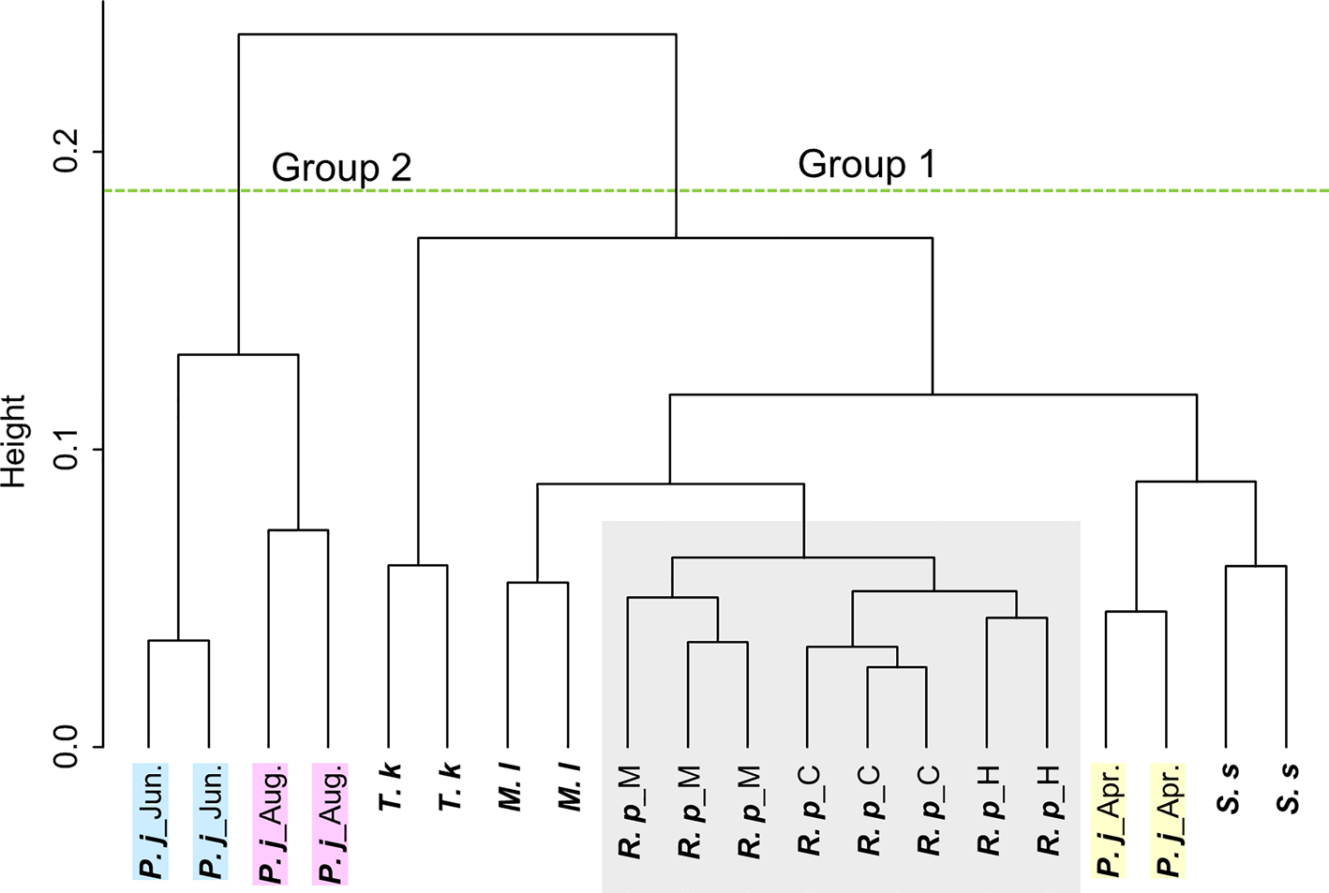
Cluster analysis based on the concentrations of amino acids detected in the tissue of bivalves. The abbreviations refer to Fig. 2. Marked in light gray are ***R. p***. and ***P. j***. April, June, and August are marked in yellow, light blue, and pink, respectively.

The results of the cluster analysis are shown in Fig. 5 (for the siphon) and Supplementary Figs. S7a–S7e (for the foot, adductor muscle, mantle, gill, and viscera, respectively). The results of the SIMPER analysis are presented in Supplementary Table S4. The siphon of *P. japonica* in April was clustered separately than those of June and August that were grouped together in a separate cluster (*p* = 0.002). Gly, l-Leu, l-Glu, and l-Phe were the main contributors to this grouping. Significant groupings were also observed in the adductor muscle (*p* = 0.001), foot (*p* = 0.001), and mantle (*p* = 0.001). The contribution of amino acids to these grouping was similar to that of the siphon. The grouping of viscera (*p* = 0.058) and gills (*p* = 0.066) exhibited a lower dissimilarity compared to the other four tissues, however, both tissues exhibited significant grouping. *R. philippinarum* samples from three regions in Japan were also classified into the same cluster across all tissues except for viscera.

### AR activity of *P. japonica* tissue

**Fig. 6:**
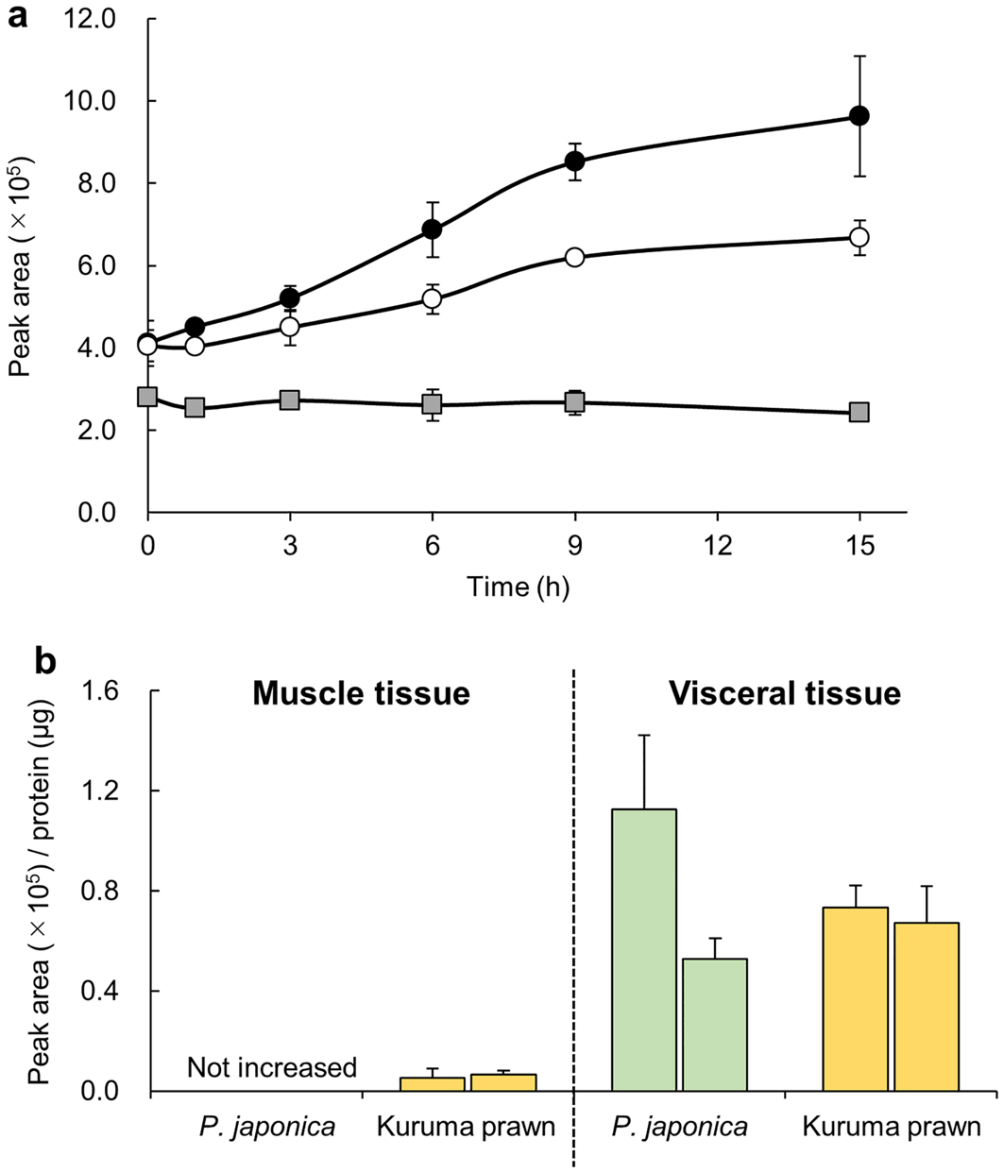
(**a**) Time-course of d-Ala^13^C,*d*_1_ production during the reaction using homogenates derived from the viscera tissue of *P. japonica* (*n* = 3 for each sample). PBS was used as a control and showed no significant increase in d-Ala^13^C,*d*_1_ production over 15 h. In contrast, the circles represent the time-course data for the homogenates prepared from the viscera tissue of two independent individuals (crude enzyme extracts), which showed a gradual increase in d-Ala^13^C,*d*_1_ levels, stabilizing at approximately 15 h. (**b**) Comparative analysis of d-Ala^13^C,*d*_1_ production after a 15-h reaction using homogenates from the muscle and viscera tissues of *P. japonica* and kuruma prawn. The viscera tissues consistently exhibited considerably higher production rates compared with the muscle tissues, clearly highlighting the enzymatic activity differences between these tissues.

Figure 6a shows the changes in the peak area of d-Ala^13^C,*d*_1_ with the addition of PBS or the homogenate of *P. japonica* viscera. The peak area of d-Ala^13^C,*d*_1_ did not exhibit any change after 15 h when PBS was added as a control experiment. In contrast, when the homogenate derived from *P. japonica* viscera was added, the amount of d-Ala^13^C,*d*_1_ began to increase after 6 h and reached a plateau after approximately 15 h. When the reaction time was fixed at 15 h and the homogenates from the muscle tissues (*P. japonica* siphon and kuruma prawn muscle) and viscera tissues (*P. japonica* viscera and kuruma prawn hepatopancreas) were added, the peak area increased more with the viscera tissues than with the muscle tissues (Fig. 6b). Furthermore, the individual differences and measurement errors were larger for *P. japonica* viscera than for kuruma prawn hepatopancreas.

### DAO activity in *P. japonica* tissue

**Fig. 7:**
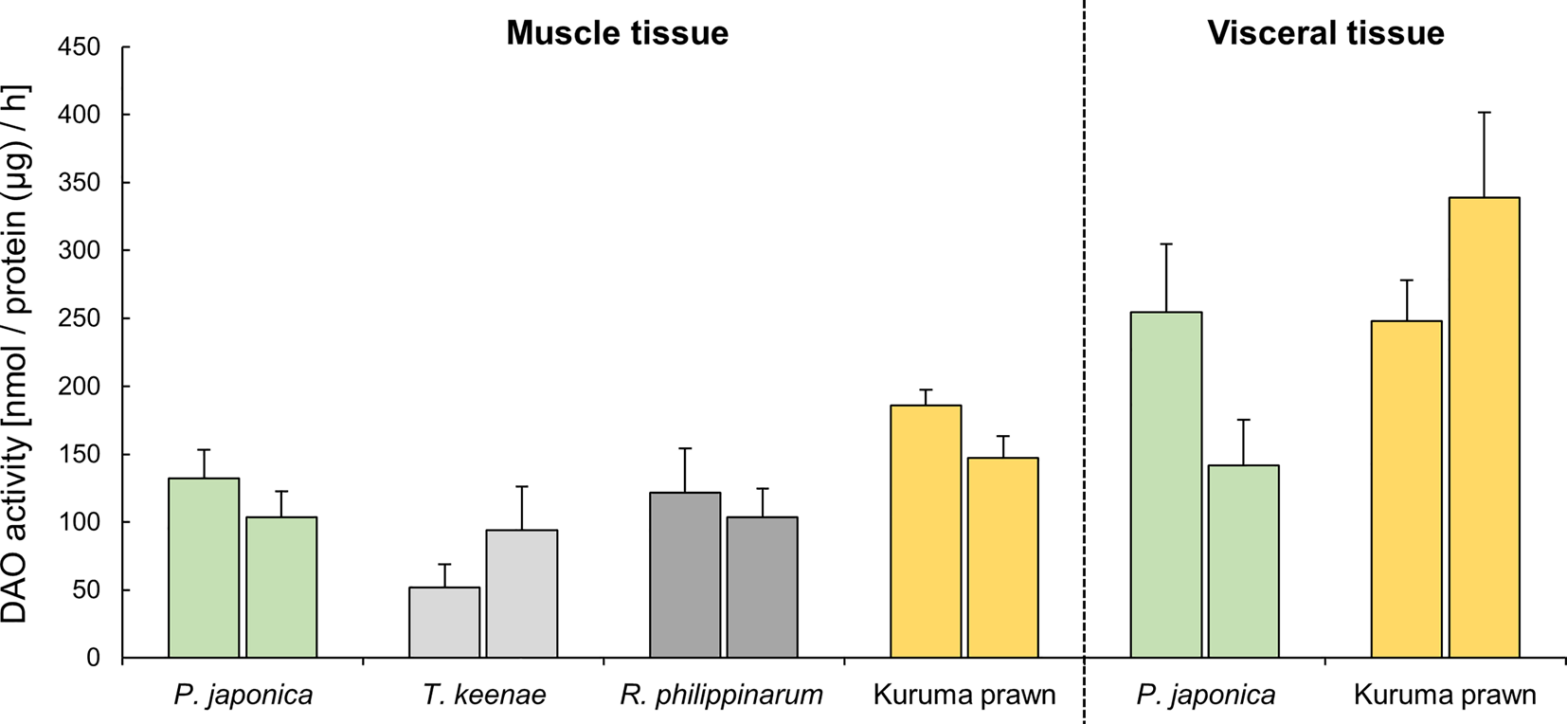
DAO activity in the muscle and viscera tissues (nmol/protein (µg)/h). Data are expressed as mean ± SD of triplicate measurements. Left side, muscle; right side, viscera tissue.

The DAO activities of muscle tissues (*P. japonica* siphon tissue and kuruma prawn muscle) and viscera tissues (*P. japonica* viscera and kuruma prawn hepatopancreas) are shown in Fig. 7. The kuruma prawn samples exhibited the highest DAO activity (nmol/protein (µg)/h) at 147–186. The DAO activity decreased in the following order: *P. japonica* (104–132), *R. philippinarum* (104–122), and *T. keenae* (52–94). The DAO activity (nmol/protein (µg)/h) of the viscera tissues was 142–255 for *P. japonica* and 248–339 for kuruma prawn, both of which were higher than that of muscle tissues.

## Discussion

In this study, we measured the d-amino acid content in *P. japonica* and identified several important d-amino acids, d-Ala, d-Pro, and d-Asp. Okuma et al.^9^. studied the d-amino acid concentrations in bivalves, however, they investigated a limited number of amino acids and *P. japonica* was not included. Furthermore, Qin et al.^13^ studied the dietary components in the siphon of *P. japonica*, however, they did not perform separate d- and l-amino acid analyses. In this study, we revealed very high concentrations of d-Ala in the siphon of *P. japonica*, and detected other d-amino acids such as d-Pro in the adductor muscle and d-Asp in the gills. Furthermore, this is the first study to reveal that the concentration of d-Ala in the siphon of *P. japonica* (6.99–14.2 mmol/100 g-wet) and its percentage, 91–94% of total Ala (d-Ala + l-Ala), considerably exceeded those of other bivalves, including *T. keenae* (74%) and *R. philippinarum* (23–41%).

Cluster analysis based on the amino acid profiles revealed specific trends associated with seasonal changes in *P. japonica*. The siphon, foot, adductor muscle, and viscera of *P. japonica* collected in “April” and “June and August” were divided into two different groups. Qin et al. reported that the concentrations of glycogen and protein in the siphon of *P. japonica* are related to the maturation of individuals, with both concentrations increasing as maturation progresses and decreasing following the release of gametophytes^13^. Therefore, the grouping of *P. japonica* by collection month in this study may also be related to reproduction. In the Sea of Japan, the reproductive season of *P. japonica* is from May to October, with the highest fertilization rate occurring between May and June^24^. In contrast, in the Seto Inland Sea around 2002, *P. japonica* matured from November to December and release eggs and sperm in January^23^. Thus, while the spawning season in the Sea of Japan occurs earlier, around October, the spawning season in the Seto Inland Sea, particularly in the Suo Sea located at the western end of the Seto Inland Sea, occurs during winter. Although simple comparisons are difficult due to differences in the environments and research periods between the Sea of Japan and the Seto Inland Sea, it is suggested that environmental factors such as sea water temperature influence the spawning season and frequency of spawning in *P. japonica*. Information regarding the spawning period of *P. japonica* in Aichi Prefecture is not available in this study, however, seasonal changes in nutrient composition—such as changes in the species and abundance of plankton consumed by *P. japonica* (noting that certain diatom species in seawater exhibit AR activity^25^)—and physiological changes related to the growth stage of individuals may be influenced by environmental factors. Given that body composition likely varies depending on the season and collection site, future research should include year-round sampling across multiple locations, taking into account the maturation stage of *P. japonica*. Notably, although the *R. philippinarum* samples were collected from three different regions, the amino acid profiles of the five tissues (excluding the viscera) were similar and classified in the same group in the cluster analysis. This suggests that adaptation to their respective living environments resulted in similar amino acid compositions.

Regarding the Gly concentrations detected in *R. philippinarum* and *P. japonica* in relation to their lifestyles, *P. japonica* exhibited a high concentration of d-Ala and particularly low Gly levels in the muscle tissue samples, whereas Gly was the most abundant amino acid in *R. philippinarum*. In adult *P. japonica*, the foot becomes degenerated, thus limiting its burrowing ability^26^, while *R. philippinarum* maintains an active foot function^27^ and well-developed adductor muscle to adapt to environmental changes. Investigating the direct relationship between muscle Gly concentrations and burrowing activity in bivalves is important for understanding their ecological and metabolic adaptations.

The AR activity for d-Ala biosynthesis was clearly observed in *P. japonica*, with the d-Ala^13^C,*d*_1_ production gradually increasing from 6 h onwards and exhibiting a plateau at 15 h in the presence of tissue homogenate. Such a result indicates that *P. japonica* actively synthesizes d-Ala for their lives. The AR activity was higher in the viscera tissue than in the muscle tissues. This tissue-specific trend was consistent with previous findings in kuruma prawn samples^28^. AR is present not only in kuruma prawns^29^ but also in other aquatic organisms such as brackish-water bivalve^30^ and crayfish^31^, where it converts l-Ala to d-Ala for osmoregulation.

Considering that *P. japonica* inhabits areas from subtidal zones to approximately 50 m in depth^11^, it may not require the strict osmoregulation necessary for species like *R. philippinarum* and *M. lusoria* that live in estuarine areas. Therefore, the high d-Ala concentration in *P. japonica* is more likely attributable to Ala production *via* the Ala-glucose cycle rather than being related to osmoregulation. In this pathway, glucose is converted to pyruvate, which is then converted to Ala *via* Ala aminotransferase^32,33^.

Although this study is limited to specific tissues and a single sampling period, it revealed that *P. japonica* exhibits a distinctive amino acid composition, reflecting unique metabolic characteristics that are not observed in other bivalves. These findings provide new insights into ecological research and highlight the prospect of *P. japonica* as a food ingredient.

To determine whether the variations in the d-Ala levels are regulated by enzymatic activity, future studies should investigate the seasonal dynamics of the AR and DAO activities across various tissues. Moreover, elucidating the mechanisms underlying tissue-specific differences in d-Ala content, such as variations in the metabolic activity or environmental influences, is essential for understanding the physiological significance of d-Ala accumulation in *P. japonica*. Furthermore, although AR activity was detected in the viscera of *P. japonica*, the possibility that this activity may be partially or entirely derived from symbiotic or commensal microorganisms cannot be excluded. To clarify the source of enzymatic activity, future studies employing microbiome analyses are essential. Additionally, while d-Ala synthesized in the viscera may be transported to the siphon, the transport route and cellular mechanisms remain unclear and should be addressed in future research.

Regarding the AR genes in *P. japonica*, although AR activity has been functionally confirmed in various aquatic species, the corresponding gene sequences related to Ala biosynthesis and metabolism remain largely uncharacterized, especially in invertebrates. While the AR gene has been identified in *Homo sapiens* (Gene ID: 63826) and *Rattus norvegicus* (Gene ID: 303306), only AR-like genes have been reported in bivalves, such as *Mya arenaria* (Gene IDs: 128223361, 128222010, and 128203193), *Dreissena polymorpha* (Gene IDs: 127866498 and 127866504), *R. philippinarum* (Gene ID: 132731142), and *Mercenaria mercenaria* (Gene ID: 123564294). Similarly, the DAO gene has been identified in mammals such as *H. sapiens* (Gene ID: 1610), *Mus musculus* (Gene ID: 13142), and *Sus scrofa* (Gene ID: 397134), while DAO-like genes have been found in bivalves, such as *Mytilus edulis* (Gene ID: 139522859), *Saccostrea cuccullata* (Gene ID: 134230625), and *Crassostrea virginica* (Gene ID: 111108560)(NCBI: http://www.ncbi.nlm.nih.gov/gene, accessed on June 29, 2025). Future studies should focus on elucidating the gene sequences of AR and DAO in *P. japonica* to advance our understanding on the differences in their primary structures compared to those in mammals, as well as on their physiological roles and seasonal variation of their expression levels in this species.

## Conclusion

This study revealed that *P. japonica* possesses an exceptionally high concentration of d-Ala in its siphon tissue, exceeding levels previously reported in other bivalves. The unique sweetness of d-Ala further highlights its potential as a culinary delicacy, particularly in dishes like *sashimi*. Our findings provide new insights into the metabolic characteristics of *P. japonica* and underscore its promising potential for food applications. Moreover, the presence of d-Ala raises important questions regarding the biological roles of d-amino acids in bivalves. Future studies should investigate the seasonal and environmental factors influencing the d-Ala concentrations, as well as the metabolic pathways involved in its synthesis and degradation. Furthermore, research into the physiological roles of d-amino acids, such as their involvement in osmoregulation and stress responses, is essential.

## Electronic supplementary material

Below is the link to the electronic supplementary material.

Supplementary Material 1

Supplementary Material 2

Supplementary Material 3

## Data Availability

All data analyzed during this study are included in this published article and its Supplementary Information files.
