# Supplementary material for: d-Alanine content in the marine edible bivalve Panopea japonica and evaluation of its associated enzyme activities
Source: Sci Rep. 2025 Jul 14;15:25415. doi: 10.1038/s41598-025-10379-2 (PMC12259925; doi:10.1038/s41598-025-10379-2)
Supplement: Supplementary file 1 — Supplementary Material 1 [file 41598_2025_10379_MOESM1_ESM.pdf]

D-Alanine content in the marine edible bivalve *Panopea japonica* and evaluation of its associated enzyme activities

Mayu Onozato[1*], Tomoya Takaura[1], Wataru Shinohara[2], Takehiro Tsukada[3], Tatsuya Sakamoto[1], Kenji Okoshi[4, 5], Takeshi Fukushima[1]

[1] *Department of Analytical Chemistry, Faculty of Pharmaceutical Sciences, Toho University, 2-2-1 Miyama, Funabashi-shi, Chiba 274-8510, Japan*

[2] *Chiba Municipal Chiba High School, 9-46-1 Konakadai, Inage-ku, Chiba-shi, Chiba 263-0043, Japan*

[3] *Department of Biomolecular Science, Faculty of Science, Toho University, 2-2-1 Miyama, Funabashi-shi, Chiba 274-8510, Japan*

[4] *Department of Environmental Science, Faculty of Science, Toho University, 2-2-1 Miyama, Funabashi-shi, Chiba 274-8510, Japan*

[5] *Toyo Institute of Food Technology, 4-23-2 Minami-Hanayashiki, Kawanishi-shi, Hyogo 666-0026, Japan*

[*]**Correspondence:** Mayu Onozato, Ph.D.

Department of Analytical Chemistry, Faculty of Pharmaceutical Sciences, Toho University, 2-2-1 Miyama, Funabashi-shi, Chiba 274-8510, Japan

Tel./Fax: 81-47-472-1523; *E-mail address:* mayu.onozato@phar.toho-u.ac.jp

Contents

1. Chemicals and reagents Page 3

2. D-Ala^{13}C,d_1 analysis Page 4

3. DAO fraction preparation Page 4

4. HPLC-fluorescence detection for MeS-KYNA Page 5

References

1. Chemicals and reagents

L-Alanine (Ala), L-arginine (Arg), L-aspartic acid (Asp), L-glutamine (Gln), L-glutamate (Glu), glycine (Gly), L-histidine (His), L-isoleucine (Ile), L-leucine (Leu), L-lysine (Lys), L-methionine (Met), L-ornithine (Orn), L-phenylalanine (Phe), L-proline (Pro), L-serine (Ser), L-tryptophan (Trp), L-tyrosine (Tyr), and L-valine (Val) were obtained from Kyowa Hakko Bio (Tokyo, Japan). D-Ala, D-Phe, D-Trp, D-Ser, high-performance liquid chromatography (HPLC)-grade CH_3OH (MeOH), liquid chromatography-mass spectrometry (LC-MS)-grade MeOH, HPLC-grade formic acid, and the APDSTAG® Wako Amino Acids Internal Standard Mixture Solution were obtained from FUJIFILM Wako Pure Chemical (Osaka, Japan). 4-Dimethylaminopyridine (DMAP), triphenylphosphine (TPP), 2,2′-dipyridyl disulfide (DPDS), β-Ala, D-Arg, D-Asp, D-Gln, D-Glu, D-His, D-Ile, D-Leu, D-Lys, and D-Val were purchased from Tokyo Chemical Industry (Tokyo, Japan). γ-Aminobutyric acid (GABA), D-Met, D-Tyr, DL-Orn, D-Pro, and ammonium formate were procured from Sigma-Aldrich (St. Louis, MO, USA). HPLC-grade CH_3CN and LC-MS-grade CH_3CN were obtained from Kanto Kagaku (Tokyo, Japan). Phosphate-buffered saline (PBS) was purchased from Nissui (Tokyo, Japan). The water used was purified using a Milli-Q Lab system (Nihon Millipore, Tokyo, Japan). Millex®-LG filters (0.20 μm) were purchased from Merck (Darmstadt, Germany).

2. D-Ala$^{13}C,d_1$ analysis

The thawed supernatant (10 μL) was mixed with 20 mM (*R*)-CIMa-OSu in CH_3CN (10 μL) and 30 mM DMAP in CH_3CN (10 μL). The solution was vortexed for 1 min and allowed to stand at room temperature (approximately 22 °C) for 60 min. Subsequently, a 0.1% formic acid in CH_3CN (70 μL) was added to stop the derivatization reaction. Then, 100 μL of the mobile phase (A:B = 9:1 (*v*/*v*), consisting of A: H_2O/MeOH/10 mM ammonium formate in H_2O (pH 2.8) (50:20:30, *v*/*v*/*v*) and B: 10 mM ammonium formate in H_2O/MeOH (30:70, *v*/*v*) was added. After vortexing for 1 min, the solution was filtered using a Millex®-LG filter (0.20 μm) and analyzed via LC–MS/MS [1].

3. DAO fraction preparation

Ice-cold phosphate-buffered saline (PBS, 1.0 mL per 100 mg sample) containing a protease inhibitor cocktail (Sigma-Aldrich, 1.0 vol%) was added to the sample, which was then homogenized using a ShakeMan 6 instrument (Biomedical Science, Tokyo, Japan) with a 6- or 10-mm stainless steel bead. Homogenization was performed for 30 sec at 4,350 rpm, followed by a 5-sec rest, and this cycle was repeated once more. The resulting suspension was centrifuged at 600 × *g* for 10 min at 4 °C. The supernatant was subsequently transferred to another tube and centrifuged at 20,000 × *g* for 20 min at 4 °C. The supernatant was discarded and the pellet was resuspended in 1.0 mL of ice-cold PBS. This suspension was centrifuged at 600 × *g* for 5 min at 4 °C, and the supernatant was used as the DAO fraction [2].

4. HPLC-fluorescence detection of MeS-KYNA

A 10-μL aliquot of each sample was injected into the HPLC system (Jasco Corporation Tokyo, Japan) equipped with an intelligent pump (PU-4180), autosampler (AS-4050i), column oven (CO-2065 *Plus*), fluorescence detector (FP-4025), and interface (LC-NetII/ADC), all controlled by ChromNavi® software. Chromatographic separation was achieved using an InertSustain® C18 column (5.0 μm, 4.6 × 250 mm; GL Sciences Inc., Tokyo, Japan), which was maintained at 40 °C. The mobile phases were 20 mM ammonium formate in H_2O/MeOH (90/10, *v*/*v*) (A) and MeOH (B) and the flow rate was 1.0 mL/min. The gradient program was as follows: 0–25 min, 20% B; 25–35 min, 100% B; 35–45 min, 20% B. Fluorescence detection was performed at an emission wavelength of 461 nm with an excitation wavelength of 363 nm.

References

[1] Onozato, M., Nakanoue, H., Sakamoto, T., Umino, M. & Fukushima, T. Determination of D- and L-amino acids in garlic foodstuffs by liquid chromatography-tandem mass spectrometry. *Molecules* **28**, 1773 (2023).

[2] Onozato, M. *et al.* Effect of risperidone on plasma D-serine concentration in rats post-administered with D-serine. *Life Sci.* **158**, 98-103 (2016).
