# Supplementary material for: d-Alanine content in the marine edible bivalve Panopea japonica and evaluation of its associated enzyme activities
Source: Sci Rep. 2025 Jul 14;15:25415. doi: 10.1038/s41598-025-10379-2 (PMC12259925; doi:10.1038/s41598-025-10379-2)
Supplement: Supplementary file 2 — Supplementary Material 2 [file 41598_2025_10379_MOESM2_ESM.pdf]

**Table S1** Measurements of the samples used in the study.

| | Month / site | *n* | Length (mm) | width (mm) | Height (mm) | Total weight (g) |
|---|---|---|---|---|---|---|
| *Panopea japonica* | Apr. / Aichi Prefecture | 2 | 108 | 65 | 30 | 305 |
| | | | 108 | 59 | 33 | 241 |
| | Jun. / Aichi Prefecture | 2 | 116 | 68 | 57 | 330 |
| | | | 111 | 69 | 56 | 300 |
| | Aug. / Aichi Prefecture | 2 | 128 | 66 | 57 | 380 |
| | | | 108 | 64 | 51 | 330 |
| *Tresus keenae* | Apr. / Aichi Prefecture | 2 | 115 | 80 | 49 | 253 |
| | | | 113 | 75 | 45 | 224 |
| *Spisula sachalinensis* | May / Miyagi Prefecture | 2 | 87 | 70 | 51 | 170 |
| | | | 78 | 64 | 47 | 150 |
| *Meretrix lusoria* | May / Miyagi Prefecture | 2 | 63 | 52 | 30 | 58 |
| | | | 54 | 46 | 28 | 30 |
| *Ruditapes philippinarum* | May / Miyagi Prefecture | 3 | 47 | 32 | 24 | 23 |
| | | | 47 | 33 | 25 | 23 |
| | | | 49 | 33 | 23 | 24 |
| | June / Hokkaido | 2 | 60 | 42 | 28 | 48 |
| | | | 58 | 44 | 30 | 48 |
| | June / Chiba Prefecture | 3 | 32 | 22 | 15 | 11 |
| | | | 32 | 21 | 13 | 8 |
| | | | 26 | 20 | 14 | 8 |
| Kuruma prawn | July / Oita Prefecture | 3 | | | | 31 |
| | | | | | | 32 |
| | | | | | | 29 |

**Table S2a** Concentrations of amino acids in the siphon of bivalves (mean ± SD [mmol/100 g-wet]).

| | ***P. j*** _Apr. | ***P. j*** _Jun. | ***P. j*** _Arg. | ***T. k*** | ***S. s*** | ***M. l*** | ***R. p*** _M | ***R. p*** _H | ***R. p*** _C |
|---|---|---|---|---|---|---|---|---|---|
| L-Gln | 0.0545 ± 0.0093 | 0.0121 ± 0.0025 | 0.0158 ± 0.0040 | 0.224 ± 0.037 | 0.0534 ± 0.012 | 0.194 ± 0.010 | 0.144 ± 0.045 | 0.131 ± 0.064 | 0.129 ± 0.021 |
| β-Ala | N.D. | N.D. | N.D. | 0.000486 ± 0.00069 | N.D. | 0.0109 ± 0.015 | 0.0259 ± 0.023 | 0.0147 ± 0.017 | 0.00964 ± 0.0086 |
| D-Ala | 14.2 ± 1.1 | 9.16 ± 1.6 | 6.99 ± 0.50 | 4.98 ± 1.2 | 2.88 ± 0.60 | 1.14 ± 0.71 | 0.214 ± 0.031 | 0.799 ± 0.22 | 0.267 ± 0.061 |
| L-Ala | 1.33 ± 0.635 | 0.532 ± 0.057 | 0.638 ± 0.15 | 1.750 ± 0.026 | 0.762 ± 0.11 | 1.18 ± 0.0028 | 0.723 ± 0.14 | 1.14 ± 0.19 | 0.657 ± 0.091 |
| L-Ser | 0.165 ± 0.0057 | 0.0360 ± 0.000087 | 0.0350 ± 0.0069 | 0.551 ± 0.17 | 0.199 ± 0.025 | 0.305 ± 0.10 | 0.201 ± 0.052 | 0.144 ± 0.002 | 0.102 ± 0.017 |
| GABA | N.D. | N.D. | N.D. | 0.00316 ± 0.0026 | 0.0115 ± 0.0067 | 0.00059 ± 0.0008 | N.D. | N.D. | N.D. |
| Gly | 0.113 ± 0.019 | 0.0559 ± 0.040 | N.D. | 4.49 ± 2.1 | 0.599 ± 0.21 | 2.92 ± 1.3 | 2.60 ± 1.7 | 4.88 ± 2.2 | 1.91 ± 1.0 |
| L-Glu | 0.0533 ± 0.010 | N.D. | 1.70 ± 0.77 | 0.269 ± 0.029 | 0.0627 ± 0.024 | 0.266 ± 0.0071 | 0.180 ± 0.072 | 0.119 ± 0.051 | 0.119 ± 0.015 |
| D-Pro | 0.0238 ± 0.012 | N.D. | N.D. | 0.0554 ± 0.034 | 0.00268 ± 0.0022 | N.D. | N.D. | N.D. | N.D. |
| L-Pro | 0.0619 ± 0.010 | 0.0146 ± 0.00035 | 0.0200 ± 0.0041 | 0.0611 ± 0.015 | 0.0476 ± 0.016 | 0.129 ± 0.074 | 0.0455 ± 0.010 | 0.0375 ± 0.0027 | 0.0296 ± 0.0037 |
| L-Val | 0.0783 ± 0.017 | 0.0287 ± 0.0019 | 0.0314 ± 0.013 | 0.0265 ± 0.0046 | 0.105 ± 0.037 | 0.231 ± 0.10 | 0.112 ± 0.0012 | 0.0979 ± 0.010 | 0.109 ± 0.022 |
| L-Met | 0.0896 ± 0.036 | 0.0111 ± 0.0011 | 0.0152 ± 0.010 | 0.0209 ± 0.0041 | 0.123 ± 0.0060 | 0.247 ± 0.037 | 0.132 ± 0.015 | 0.0744 ± 0.017 | 0.0644 ± 0.0052 |
| L-His | 0.0324 ± 0.011 | 0.0212 ± 0.0008 | 0.0210 ± 0.002 | 0.0239 ± 0.0025 | 0.0310 ± 0.00037 | 0.0700 ± 0.025 | 0.0441 ± 0.0056 | 0.0554 ± 0.0035 | 0.0374 ± 0.0039 |
| D-Asp | 0.102 ± 0.008 | 0.0236 ± 0.0061 | 0.0619 ± 0.011 | 0.00763 ± 0.0026 | 0.00770 ± 0.0036 | 0.0532 ± 0.022 | 0.0150 ± 0.020 | 0.0136 ± 0.0056 | 0.00718 ± 0.0040 |
| L-Asp | 0.170 ± 0.029 | 0.106 ± 0.030 | 0.173 ± 0.032 | 0.290 ± 0.29 | 0.268 ± 0.23 | 0.424 ± 0.0010 | 0.752 ± 0.28 | 0.121 ± 0.062 | 0.417 ± 0.11 |
| L-Ile | 0.0776 ± 0.012 | 0.00774 ± 0.0019 | 0.0154 ± 0.010 | 0.0127 ± 0.0063 | 0.0944 ± 0.017 | 0.240 ± 0.087 | 0.100 ± 0.0045 | 0.0455 ± 0.0048 | 0.0625 ± 0.011 |
| L-Leu | 0.159 ± 0.021 | 0.0006 ± 0.00012 | 0.00699 ± 0.010 | 0.0143 ± 0.018 | 0.174 ± 0.024 | 0.537 ± 0.17 | 0.207 ± 0.011 | 0.0768 ± 0.013 | 0.112 ± 0.015 |
| L-Arg | 3.18 ± 0.92 | 0.468 ± 0.14 | 0.622 ± 0.22 | 5.89 ± 1.37 | 1.45 ± 0.98 | 0.757 ± 0.42 | 1.13 ± 0.55 | 0.439 ± 0.025 | 0.587 ± 0.090 |
| L-Trp | 0.0113 ± 0.00030 | 0.0111 ± 0.00083 | 0.00930 ± 0.0014 | 0.00762 ± 0.00048 | 0.0168 ± 0.0018 | 0.0379 ± 0.016 | 0.0236 ± 0.0060 | 0.0375 ± 0.0093 | 0.0335 ± 0.0025 |
| L-Phe | 0.0343 ± 0.0052 | N.D. | 0.00144 ± 0.0020 | N.D. | 0.0777 ± 0.0041 | 0.263 ± 0.060 | 0.100 ± 0.0047 | 0.0385 ± 0.015 | 0.0684 ± 0.017 |
| L-Orn | 0.0153 ± 0.022 | N.D. | N.D. | N.D. | 0.0119 ± 0.017 | N.D. | N.D. | N.D. | N.D. |
| L-Lys | 0.209 ± 0.023 | 0.0131 ± 0.0064 | 0.0195 ± 0.0084 | 0.114 ± 0.019 | 0.222 ± 0.039 | 0.395 ± 0.18 | 0.255 ± 0.057 | 0.0927 ± 0.0064 | 0.107 ± 0.039 |
| L-Tyr | 0.0782 ± 0.016 | 0.0261 ± 0.0023 | 0.0393 ± 0.012 | 0.0328 ± 0.0045 | 0.131 ± 0.018 | 0.263 ± 0.066 | 0.175 ± 0.040 | 0.0744 ± 0.011 | 0.106 ± 0.0068 |

**Table S2b** Concentrations of amino acids in the foot of bivalves (mean ± SD [mmol/100 g-wet]).

| | ***P. j***_Apr. | ***P. j***_Jun. | ***P. j***_Arg. | ***T. k*** | ***S. s*** | ***M. l*** | ***R. p***_M | ***R. p***_H | ***R. p***_C |
|---|---|---|---|---|---|---|---|---|---|
| L-Gln | 0.0795 ± 0.019 | 0.0107 ± 0.00012 | 0.0206 ± 0.000077 | 0.249 ± 0.016 | 0.0428 ± 0.015 | 0.0825 ± 0.038 | 0.111 ± 0.043 | 0.0424 ± 0.0028 | 0.1799 ± 0.15 |
| β-Ala | 0.00447 ± 0.0063 | N.D. | | 0.0541 ± 0.037 | 0.00931 ± 0.013 | 0.00610 ± 0.0086 | 0.011 ± 0.0094 | 0.0370 ± 0.016 | 0.0289 ± 0.011 |
| D-Ala | 2.92 ± 0.40 | 2.32 ± 0.15 | 2.54 ± 0.23 | 0.689 ± 0.11 | 0.510 ± 0.21 | 0.953 ± 0.54 | 0.525 ± 0.19 | 0.989 ± 0.21 | 0.3695 ± 0.25 |
| L-Ala | 0.477 ± 0.082 | 0.463 ± 0.042 | 0.440 ± 0.012 | 1.42 ± 0.037 | 1.15 ± 0.67 | 1.34 ± 0.33 | 1.03 ± 0.19 | 1.42 ± 0.13 | 0.9080 ± 0.23 |
| L-Ser | 0.0991 ± 0.017 | 0.0406 ± 0.0045 | 0.0360 ± 0.0074 | 0.263 ± 0.012 | 0.190 ± 0.053 | 0.156 ± 0.04 | 0.120 ± 0.022 | 0.106 ± 0.0030 | 0.0745 ± 0.010 |
| GABA | N.D. | N.D. | 0.00120 ± 0.00080 | 0.00433 ± 0.0030 | 0.0177 ± 0.018 | 0.000983 ± 0.00030 | N.D. | N.D. | N.D. |
| Gly | 0.8580 ± 0.078 | 0.605 ± 0.12 | 0.417 ± 0.023 | 7.71 ± 0.034 | 3.52 ± 2.0 | 0.233 ± 0.21 | 4.92 ± 1.1 | 6.09 ± 1.2 | 2.6375 ± 1.4 |
| L-Glu | 0.0708 ± 0.043 | 0.00181 ± 0.0026 | 2.39 ± 0.79 | 0.290 ± 0.019 | 0.0534 ± 0.0087 | 0.148 ± 0.041 | 0.132 ± 0.060 | 0.0368 ± 0.0061 | 0.1637 ± 0.14 |
| D-Pro | 0.00718 ± 0.0052 | N.D. | | 0.0246 ± 0.0070 | 0.00930 ± 0.013 | N.D. | N.D. | N.D. | N.D. |
| L-Pro | 0.0525 ± 0.010 | 0.0161 ± 0.00011 | 0.0217 ± 0.0015 | 0.0440 ± 0.0077 | 0.0426 ± 0.0071 | 0.0825 ± 0.028 | 0.0362 ± 0.015 | 0.0374 ± 0.0034 | 0.0315 ± 0.0032 |
| L-Val | 0.0561 ± 0.013 | 0.0280 ± 0.0026 | 0.0338 ± 0.0044 | 0.0283 ± 0.0023 | 0.0727 ± 0.0071 | 0.159 ± 0.022 | 0.0664 ± 0.0073 | 0.0669 ± 0.016 | 0.0758 ± 0.0056 |
| L-Met | 0.0484 ± 0.024 | 0.00929 ± 0.0023 | 0.0126 ± 0.0018 | 0.0290 ± 0.0023 | 0.0707 ± 0.023 | 0.181 ± 0.034 | 0.0716 ± 0.0079 | 0.0502 ± 0.021 | 0.0399 ± 0.014 |
| L-His | 0.0708 ± 0.056 | 0.0484 ± 0.0049 | 0.0376 ± 0.0061 | 0.0278 ± 0.0069 | 0.0345 ± 0.015 | 0.0496 ± 0.018 | 0.0411 ± 0.0063 | 0.0616 ± 0.0095 | 0.0347 ± 0.0061 |
| D-Asp | 0.0935 ± 0.032 | 0.0352 ± 0.0087 | 0.0676 ± 0.0012 | 0.00950 ± 0.0065 | 0.0241 ± 0.030 | 0.00831 ± 0.0090 | N.D. | N.D. | 0.0007 ± 0.0012 |
| L-Asp | 1.33 ± 0.012 | 0.169 ± 0.026 | 0.441 ± 0.084 | 0.646 ± 0.13 | 0.0828 ± 0.020 | 0.240 ± 0.11 | 0.664 ± 0.21 | 0.0933 ± 0.040 | 0.5194 ± 0.47 |
| L-Ile | 0.0408 ± 0.0057 | 0.00614 ± 0.0018 | 0.0103 ± 0.00034 | 0.0186 ± 0.0032 | 0.0635 ± 0.015 | 0.158 ± 0.0075 | 0.0491 ± 0.010 | 0.0261 ± 0.0082 | 0.0308 ± 0.0054 |
| L-Leu | 0.0623 ± 0.012 | 0.000445 ± 0.00063 | 0.0112 ± 0.0077 | 0.0247 ± 0.0045 | 0.108 ± 0.038 | 0.382 ± 0.019 | 0.0937 ± 0.023 | 0.0479 ± 0.022 | 0.0569 ± 0.011 |
| L-Arg | 0.859 ± 0.079 | 0.136 ± 0.028 | 0.248 ± 0.017 | 6.87 ± 0.30 | 2.48 ± 2.2 | 0.951 ± 0.93 | 1.14 ± 0.38 | 0.602 ± 0.027 | 0.5999 ± 0.011 |
| L-Trp | 0.0106 ± 0.0017 | 0.0112 ± 0.00093 | 0.00932 ± 0.00073 | 0.00874 ± 0.00020 | 0.0123 ± 0.00043 | 0.033 ± 0.010 | 0.0150 ± 0.0024 | 0.0216 ± 0.0017 | 0.0203 ± 0.0039 |
| L-Phe | 0.0142 ± 0.016 | N.D. | N.D. | N.D. | 0.0428 ± 0.015 | 0.183 ± 0.008 | 0.0321 ± 0.0040 | 0.0156 ± 0.0048 | 0.0263 ± 0.0034 |
| L-Orn | 0.00682 ± 0.0093 | N.D. | N.D. | N.D. | 0.0132 ± 0.019 | N.D. | N.D. | N.D. | 0.0033 ± 0.0030 |
| L-Lys | 0.129 ± 0.066 | 0.0200 ± 0.0020 | 0.0291 ± 0.0012 | 0.151 ± 0.0054 | 0.174 ± 0.074 | 0.180 ± 0.045 | 0.102 ± 0.058 | 0.0732 ± 0.0035 | 0.0983 ± 0.020 |
| L-Tyr | 0.0535 ± 0.01 | 0.0264 ± 0.0027 | 0.0399 ± 0.0037 | 0.0408 ± 0.0028 | 0.0848 ± 0.00037 | 0.199 ± 0.010 | 0.0848 ± 0.0046 | 0.0498 ± 0.0055 | 0.0751 ± 0.017 |

**Table S2c** Concentrations of amino acids in the addctor muscle of bivalves (mean ± SD [mmol/100 g-wet]).

| | ***P. j***_Apr. | ***P. j***_Jun. | ***P. j***_Arg. | ***T. k*** | ***S. s*** | ***M. l*** | ***R. p***_M | ***R. p***_H | ***R. p***_C |
|---|---|---|---|---|---|---|---|---|---|
| L-Gln | 0.190 ± 0.25 | 0.0112 ± 0.093 | 0.0273 ± 0.025 | 0.0663 ± 0.068 | 0.0318 ± 0.00062 | 0.0658 ± 0.040 | 0.132 ± 0.059 | 0.0334 ± 0.0066 | 0.0795 ± 0.070 |
| β-Ala | 0.00145 ± 0.0021 | N.D. | N.D. | N.D. | 0.00264 ± 0.0037 | 0.0237 ± 0.033 | 0.0184 ± 0.0060 | 0.00966 ± 0.012 | 0.00461 ± 0.0080 |
| D-Ala | 4.74 ± 3.2 | 4.72 ± 0.28 | 2.97 ± 0.11 | 2.01 ± 1.2 | 0.706 ± 0.48 | 1.10 ± 0.71 | 0.503 ± 0.14 | 1.34 ± 0.11 | 0.446 ± 0.14 |
| L-Ala | 1.07 ± 0.21 | 0.569 ± 1.2 | 0.664 ± 0.043 | 1.16 ± 0.22 | 0.883 ± 0.46 | 1.36 ± 0.31 | 0.867 ± 0.18 | 1.38 ± 0.075 | 0.840 ± 0.17 |
| L-Ser | 0.280 ± 0.10 | 0.0368 ± 0.026 | 0.0283 ± 0.0058 | 0.560 ± 0.064 | 0.158 ± 0.053 | 0.145 ± 0.040 | 0.141 ± 0.035 | 0.0916 ± 0.022 | 0.043 ± 0.00077 |
| GABA | N.D. | N.D. | 0.00159 ± 0.0005 | 0.001 ± 0.0010 | 0.00627 ± 0.0009 | 0.00183 ± 0.0013 | N.D. | N.D. | N.D. |
| Gly | 0.977 ± 0.17 | 0.501 ± 2.5 | 0.162 ± 0.0089 | 2.979 ± 1.5 | 1.56 ± 0.24 | 0.879 ± 0.34 | 10.04 ± 3.8 | 9.87 ± 0.92 | 3.39 ± 1.2 |
| L-Glu | 0.220 ± 0.30 | N.D. | 1.622 ± 0.97 | 0.0741 ± 0.084 | 0.0338 ± 0.0055 | 0.140 ± 0.031 | 0.142 ± 0.078 | 0.0218 ± 0.0098 | 0.0669 ± 0.073 |
| D-Pro | 0.0586 ± 0.062 | 0.00396 ± 0.020 | 0.0110 ± 0.0091 | 0.0315 ± 0.018 | 0.0121 ± 0.017 | N.D. | N.D. | N.D. | N.D. |
| L-Pro | 0.0750 ± 0.0010 | 0.0115 ± 0.022 | 0.0158 ± 0.0027 | 0.0489 ± 0.016 | 0.0400 ± 0.0082 | 0.0900 ± 0.0083 | 0.0296 ± 0.011 | 0.0268 ± 0.0067 | 0.0176 ± 0.0054 |
| L-Val | 0.0687 ± 0.049 | 0.0187 ± 0.027 | 0.0239 ± 0.0045 | 0.0200 ± 0.0008 | 0.0539 ± 0.0089 | 0.130 ± 0.028 | 0.0474 ± 0.015 | 0.0541 ± 0.015 | 0.0405 ± 0.0075 |
| L-Met | 0.152 ± 0.12 | 0.00921 ± 0.015 | 0.0189 ± 0.00083 | 0.0148 ± 0.0034 | 0.0615 ± 0.0074 | 0.114 ± 0.013 | 0.0591 ± 0.018 | 0.0392 ± 0.019 | 0.0175 ± 0.0031 |
| L-His | 0.0776 ± 0.055 | 0.0449 ± 0.074 | 0.0522 ± 0.0024 | 0.0168 ± 0.0077 | 0.0282 ± 0.014 | 0.0457 ± 0.012 | 0.0442 ± 0.012 | 0.0517 ± 0.015 | 0.0226 ± 0.0067 |
| D-Asp | 0.00744 ± 0.0056 | N.D. | 0.00308 ± 0.00088 | 0.00495 ± 0.0012 | 0.00345 ± 0.0037 | 0.00652 ± 0.0011 | N.D. | N.D. | N.D. |
| L-Asp | 0.141 ± 0.13 | 0.0399 ± 0.13 | 0.0513 ± 0.0015 | 0.0722 ± 0.010 | 0.187 ± 0.074 | 0.202 ± 0.014 | 0.239 ± 0.11 | 0.162 ± 0.10 | 0.112 ± 0.095 |
| L-Ile | 0.0713 ± 0.051 | 0.00280 ± 0.014 | 0.0113 ± 0.0067 | 0.00965 ± 0.00065 | 0.0436 ± 0.00064 | 0.105 ± 0.030 | 0.0317 ± 0.018 | 0.0178 ± 0.010 | 0.00980 ± 0.0038 |
| L-Leu | 0.103 ± 0.062 | N.D. | 0.00535 ± 0.0076 | 0.000757 ± 0.0011 | 0.0789 ± 0.016 | 0.223 ± 0.041 | 0.0665 ± 0.049 | 0.0202 ± 0.020 | 0.0115 ± 0.0025 |
| L-Arg | 2.30 ± 0.67 | 0.471 ± 1.3 | 0.878 ± 0.061 | 3.91 ± 1.185 | 1.84 ± 1.5 | 1.25 ± 1.1 | 1.09 ± 0.25 | 0.525 ± 0.047 | 0.374 ± 0.11 |
| L-Trp | 0.0135 ± 0.0086 | 0.00819 ± 0.018 | 0.00696 ± 0.00085 | 0.00735 ± 0.00014 | 0.0118 ± 0.00051 | 0.0248 ± 0.010 | 0.0141 ± 0.0016 | 0.0194 ± 0.0059 | 0.0158 ± 0.0019 |
| L-Phe | 0.0180 ± 0.022 | N.D. | N.D. | N.D. | 0.0269 ± 0.000071 | 0.0813 ± 0.027 | 0.0158 ± 0.016 | 0.0017 ± 0.0024 | 0.00329 ± 0.0035 |
| L-Orn | 0.0295 ± 0.021 | N.D. | N.D. | N.D. | 0.0100 ± 0.014 | N.D. | N.D. | N.D. | N.D. |
| L-Lys | 0.175 ± 0.15 | 0.0176 ± 0.026 | 0.0253 ± 0.027 | 0.113 ± 0.041 | 0.0917 ± 0.021 | 0.120 ± 0.011 | 0.0531 ± 0.056 | 0.0525 ± 0.0045 | 0.0521 ± 0.012 |
| L-Tyr | 0.0656 ± 0.054 | 0.0229 ± 0.030 | 0.0381 ± 0.015 | 0.0321 ± 0.00047 | 0.0772 ± 0.012 | 0.155 ± 0.042 | 0.0693 ± 0.013 | 0.0393 ± 0.0071 | 0.0456 ± 0.0072 |

**Table S2d** Concentrations of amino acids in the mantle of bivalves (mean ± SD [mmol/100 g-wet]).

| | ***P. j***_Apr. | ***P. j***_Jun. | ***P. j***_Arg. | ***T. k*** | ***S. s*** | ***M. l*** | ***R. p***_M | ***R. p***_H | ***R. p***_C |
|---|---|---|---|---|---|---|---|---|---|
| L-Gln | 0.0605 ± 0.056 | 0.0176 ± 0.0088 | 0.189 ± 0.075 | 0.239 ± 0.015 | 0.0551 ± 0.036 | 0.0920 ± 0.028 | 0.0814 ± 0.012 | 0.0861 ± 0.059 | 0.134 ± 0.049 |
| β-Ala | 0.00613 ± 0.0050 | N.D. | N.D. | N.D. | 0.000198 ± 0.00028 | 0.00437 ± 0.00042 | 0.0119 ± 0.0072 | 0.0218 ± 0.025 | 0.0231 ± 0.0085 |
| D-Ala | 1.45 ± 0.77 | 1.38 ± 0.88 | 2.42 ± 1.26 | 2.83 ± 0.75 | 0.668 ± 0.46 | 0.506 ± 0.032 | 0.358 ± 0.14 | 0.702 ± 0.0041 | 0.217 ± 0.11 |
| L-Ala | 0.341 ± 0.15 | 0.324 ± 0.13 | 0.678 ± 0.19 | 0.675 ± 0.17 | 0.541 ± 0.17 | 0.821 ± 0.24 | 0.742 ± 0.22 | 1.06 ± 0.039 | 0.620 ± 0.086 |
| L-Ser | 0.0825 ± 0.047 | 0.0289 ± 0.018 | 0.0787 ± 0.023 | 0.226 ± 0.020 | 0.148 ± 0.063 | 0.150 ± 0.043 | 0.122 ± 0.021 | 0.113 ± 0.025 | 0.085 ± 0.00079 |
| GABA | N.D. | N.D. | 0.00425 ± 0.0060 | 0.00366 ± 0.00022 | 0.0121 ± 0.012 | 0.000394 ± 0.00056 | N.D. | N.D. | N.D. |
| Gly | 0.309 ± 0.42 | 0.279 ± 0.33 | 0.611 ± 0.41 | 3.427 ± 0.34 | 0.823 ± 0.45 | 0.401 ± 0.038 | 2.84 ± 0.084 | 4.50 ± 0.89 | 1.34 ± 0.32 |
| L-Glu | 0.0666 ± 0.078 | 0.00574 ± 0.0081 | 2.46 ± 0.17 | 0.283 ± 0.011 | 0.0651 ± 0.048 | 0.153 ± 0.060 | 0.0877 ± 0.029 | 0.0795 ± 0.071 | 0.118 ± 0.049 |
| D-Pro | 0.0037 ± 0.0052 | N.D. | 0.00491 ± 0.00048 | 0.0514 ± 0.008 | 0.00415 ± 0.0059 | N.D. | N.D. | N.D. | N.D. |
| L-Pro | 0.0334 ± 0.027 | 0.0108 ± 0.0047 | 0.0298 ± 0.0034 | 0.0636 ± 0.0053 | 0.0397 ± 0.010 | 0.0735 ± 0.038 | 0.0399 ± 0.0060 | 0.0328 ± 0.0013 | 0.0288 ± 0.0028 |
| L-Val | 0.0540 ± 0.033 | 0.0275 ± 0.017 | 0.0475 ± 0.0089 | 0.0200 ± 0.0010 | 0.0820 ± 0.033 | 0.150 ± 0.041 | 0.0834 ± 0.0059 | 0.0757 ± 0.0027 | 0.0940 ± 0.0058 |
| L-Met | 0.0229 ± 0.013 | 0.00343 ± 0.0049 | 0.0523 ± 0.013 | 0.0191 ± 0.0010 | 0.0755 ± 0.065 | 0.137 ± 0.0022 | 0.0841 ± 0.013 | 0.0462 ± 0.0039 | 0.0492 ± 0.0063 |
| L-His | 0.0334 ± 0.028 | 0.0220 ± 0.019 | 0.135 ± 0.033 | 0.0191 ± 0.0045 | 0.0254 ± 0.012 | 0.0437 ± 0.026 | 0.0381 ± 0.012 | 0.0507 ± 0.0032 | 0.0337 ± 0.0044 |
| D-Asp | 0.0203 ± 0.0046 | 0.00712 ± 0.0013 | 0.00755 ± 0.0024 | 0.0193 ± 0.0080 | 0.0303 ± 0.035 | 0.0158 ± 0.014 | 0.00195 ± 0.0022 | N.D. | 0.00587 ± 0.0023 |
| L-Asp | 0.316 ± 0.079 | 0.109 ± 0.0043 | 0.173 ± 0.087 | 2.098 ± 0.438 | 0.461 ± 0.45 | 0.187 ± 0.037 | 0.636 ± 0.13 | 0.177 ± 0.083 | 0.501 ± 0.040 |
| L-Ile | 0.0305 ± 0.022 | 0.00425 ± 0.0060 | 0.0307 ± 0.0086 | 0.00925 ± 0.0011 | 0.0744 ± 0.046 | 0.143 ± 0.030 | 0.0702 ± 0.0076 | 0.0331 ± 0.0048 | 0.0480 ± 0.0064 |
| L-Leu | 0.0434 ± 0.043 | 0.00176 ± 0.0025 | 0.0490 ± 0.015 | 0.00795 ± 0.003 | 0.125 ± 0.094 | 0.320 ± 0.032 | 0.128 ± 0.016 | 0.0516 ± 0.0059 | 0.0843 ± 0.0091 |
| L-Arg | 0.197 ± 0.22 | 0.0101 ± 0.014 | 0.996 ± 0.33 | 2.587 ± 0.249 | 1.00 ± 0.69 | 0.482 ± 0.40 | 0.646 ± 0.25 | 0.458 ± 0.010 | 0.384 ± 0.073 |
| L-Trp | 0.00856 ± 0.0018 | 0.00905 ± 0.0021 | 0.0291 ± 0.0053 | 0.00807 ± 0.00017 | 0.0137 ± 0.0039 | 0.0286 ± 0.012 | 0.0174 ± 0.0042 | 0.0242 ± 0.0006 | 0.0252 ± 0.0012 |
| L-Phe | N.D. | N.D. | 0.0137 ± 0.0036 | N.D. | 0.0565 ± 0.054 | 0.138 ± 0.012 | 0.0483 ± 0.0070 | 0.0207 ± 0.0023 | 0.0509 ± 0.0072 |
| L-Orn | N.D. | N.D. | 0.00600 ± 0.0021 | N.D. | 0.0112 ± 0.016 | 0.0034 ± 0.0048 | N.D. | N.D. | N.D. |
| L-Lys | 0.0475 ± 0.043 | 0.00314 ± 0.0044 | 0.221 ± 0.027 | 0.116 ± 0.0088 | 0.160 ± 0.12 | 0.182 ± 0.011 | 0.103 ± 0.043 | 0.0656 ± 0.011 | 0.0895 ± 0.018 |
| L-Tyr | 0.0283 ± 0.0064 | 0.0206 ± 0.0063 | 0.120 ± 0.014 | 0.0336 ± 0.0033 | 0.0928 ± 0.043 | 0.166 ± 0.027 | 0.0975 ± 0.012 | 0.0526 ± 0.0010 | 0.0822 ± 0.0054 |

**Table S2e** Concentrations of amino acids in the gill of bivalves (mean ± SD [mmol/100 g-wet]).

| | ***P. j*** _Apr. | ***P. j*** _Jun. | ***P. j*** _Arg. | ***T. k*** | ***S. s*** | ***M. l*** | ***R. p*** _M | ***R. p*** _H | ***R. p*** _C |
|---|---|---|---|---|---|---|---|---|---|
| L-Gln | 0.179 ± 0.039 | 0.0997 ± 0.0042 | 0.0481 ± 0.0095 | 0.184 ± 0.019 | 0.0193 ± 0.0063 | 0.0400 ± 0.027 | 0.0577 ± 0.0017 | 0.0397 ± 0.0055 | 0.114 ± 0.028 |
| β-Ala | 0.224 ± 0.16 | 0.00273 ± 0.0039 | N.D. | 0.0603 ± 0.021 | 0.00179 ± 0.0025 | 0.00976 ± 0.0042 | 0.00471 ± 0.0046 | 0.000164 ± 0.00023 | 0.00946 ± 0.015 |
| D-Ala | 1.85 ± 0.33 | 2.35 ± 0.27 | 1.42 ± 0.47 | 1.34 ± 0.25 | 0.299 ± 0.058 | 0.324 ± 0.30 | 0.201 ± 0.030 | 0.241 ± 0.037 | 0.206 ± 0.13 |
| L-Ala | 1.19 ± 0.16 | 1.11 ± 0.035 | 0.838 ± 0.25 | 1.27 ± 0.25 | 0.353 ± 0.070 | 0.672 ± 0.63 | 0.742 ± 0.097 | 0.615 ± 0.068 | 0.696 ± 0.13 |
| L-Ser | 0.364 ± 0.052 | 0.177 ± 0.0087 | 0.0801 ± 0.0050 | 0.330 ± 0.016 | 0.0891 ± 0.051 | 0.105 ± 0.084 | 0.104 ± 0.018 | 0.0587 ± 0.0021 | 0.0741 ± 0.011 |
| GABA | 0.00253 ± 0.00020 | N.D. | 0.00197 ± 0.0028 | 0.0783 ± 0.034 | 0.0172 ± 0.012 | 0.000288 ± 0.00041 | N.D. | N.D. | N.D. |
| Gly | 2.06 ± 0.40 | 1.16 ± 0.30 | 0.436 ± 0.11 | 2.14 ± 0.37 | 0.615 ± 0.54 | 0.353 ± 0.50 | 1.22 ± 0.090 | 0.980 ± 0.23 | 1.32 ± 0.64 |
| L-Glu | 0.215 ± 0.044 | 0.0854 ± 0.0068 | 1.42 ± 0.58 | 0.221 ± 0.018 | 0.0229 ± 0.032 | 0.107 ± 0.012 | 0.0681 ± 0.038 | 0.0288 ± 0.0031 | 0.0952 ± 0.025 |
| D-Pro | 0.0135 ± 0.0035 | N.D. | N.D. | N.D. | N.D. | N.D. | N.D. | N.D. | N.D. |
| L-Pro | 0.189 ± 0.015 | 0.0983 ± 0.0019 | 0.0677 ± 0.0091 | 0.117 ± 0.0080 | 0.0327 ± 0.018 | 0.0760 ± 0.074 | 0.0553 ± 0.012 | 0.0392 ± 0.0024 | 0.0440 ± 0.0061 |
| L-Val | 0.226 ± 0.035 | 0.147 ± 0.0035 | 0.0745 ± 0.017 | 0.137 ± 0.028 | 0.0510 ± 0.022 | 0.121 ± 0.088 | 0.0960 ± 0.014 | 0.0709 ± 0.0069 | 0.0914 ± 0.012 |
| L-Met | 0.0963 ± 0.019 | 0.0306 ± 0.0032 | 0.0158 ± 0.020 | 0.158 ± 0.040 | 0.0285 ± 0.0047 | 0.0756 ± 0.047 | 0.0716 ± 0.018 | 0.0319 ± 0.0027 | 0.0373 ± 0.0055 |
| L-His | 0.0473 ± 0.0053 | 0.0661 ± 0.0010 | 0.0283 ± 0.021 | 0.0480 ± 0.00075 | 0.00964 ± 0.0057 | 0.0431 ± 0.040 | 0.0456 ± 0.0071 | 0.0463 ± 0.0015 | 0.0401 ± 0.0078 |
| D-Asp | 0.218 ± 0.089 | 0.102 ± 0.017 | 0.0829 ± 0.0068 | 0.624 ± 0.10 | 0.0100 ± 0.0029 | 0.0124 ± 0.0032 | 0.102 ± 0.025 | 0.0515 ± 0.016 | 0.155 ± 0.041 |
| L-Asp | 0.694 ± 0.14 | 0.282 ± 0.032 | 0.151 ± 0.0039 | 1.72 ± 0.34 | 0.220 ± 0.11 | 0.152 ± 0.12 | 0.448 ± 0.079 | 0.146 ± 0.019 | 0.408 ± 0.16 |
| L-Ile | 0.209 ± 0.039 | 0.0648 ± 0.0020 | 0.0401 ± 0.015 | 0.130 ± 0.026 | 0.0387 ± 0.014 | 0.105 ± 0.077 | 0.0792 ± 0.018 | 0.0294 ± 0.0069 | 0.0421 ± 0.0086 |
| L-Leu | 0.452 ± 0.066 | 0.142 ± 0.0010 | 0.0599 ± 0.041 | 0.328 ± 0.090 | 0.0601 ± 0.027 | 0.197 ± 0.14 | 0.151 ± 0.038 | 0.0483 ± 0.013 | 0.0714 ± 0.020 |
| L-Arg | 0.266 ± 0.0028 | 0.0726 ± 0.015 | 0.0391 ± 0.055 | 0.538 ± 0.088 | 0.181 ± 0.14 | 0.0311 ± 0.010 | 0.280 ± 0.10 | 0.114 ± 0.0087 | 0.151 ± 0.0076 |
| L-Trp | 0.0168 ± 0.00065 | 0.0254 ± 0.0019 | 0.0108 ± 0.0034 | 0.0159 ± 0.0015 | 0.00990 ± 0.00068 | 0.0227 ± 0.015 | 0.0167 ± 0.0033 | 0.0193 ± 0.0026 | 0.0207 ± 0.00087 |
| L-Phe | 0.0375 ± 0.022 | 0.00753 ± 0.0037 | 0.0119 ± 0.017 | 0.105 ± 0.034 | 0.0113 ± 0.0026 | 0.0772 ± 0.053 | 0.0509 ± 0.0080 | 0.0156 ± 0.0023 | 0.0282 ± 0.0072 |
| L-Orn | 0.136 ± 0.14 | 0.00488 ± 0.0069 | 0.00740 ± 0.010 | 0.0169 ± 0.0059 | N.D. | 0.00721 ± 0.0083 | N.D. | N.D. | N.D. |
| L-Lys | 0.272 ± 0.017 | 0.0780 ± 0.010 | 0.0520 ± 0.074 | 0.606 ± 0.15 | 0.0895 ± 0.022 | 0.131 ± 0.048 | 0.206 ± 0.14 | 0.0828 ± 0.0018 | 0.108 ± 0.024 |
| L-Tyr | 0.103 ± 0.0023 | 0.0342 ± 0.0060 | 0.0247 ± 0.0089 | 0.160 ± 0.032 | 0.0510 ± 0.019 | 0.109 ± 0.056 | 0.0939 ± 0.0055 | 0.0421 ± 0.0040 | 0.0647 ± 0.00079 |

**Table S2f** Concentrations of amino acids in the viscera of bivalves (mean ± SD [mmol/100 g-wet]).

| | ***P. j*** _Apr. | ***P. j*** _Jun. | ***P. j*** _Arg. | ***T. k*** | ***S. s*** | ***M. l*** | ***R. p*** _M | ***R. p*** _H | ***R. p*** _C |
|---|---|---|---|---|---|---|---|---|---|
| L-Gln | 0.238 ± 0.019 | 0.112 ± 0.0048 | 0.1000 ± 0.036 | 0.265 ± 0.062 | 0.0365 ± 0.024 | 0.340 ± 0.017 | 0.182 ± 0.050 | 0.173 ± 0.16 | 0.187 ± 0.047 |
| β-Ala | 0.0174 ± 0.0068 | 0.00804 ± 0.011 | 0.000814 ± 0.00027 | 0.0163 ± 0.0068 | 0.0118 ± 0.017 | 0.0358 ± 0.0058 | 0.0507 ± 0.012 | 0.0290 ± 0.028 | 0.0159 ± 0.021 |
| D-Ala | 1.25 ± 0.062 | 1.87 ± 0.133 | 1.15 ± 0.25 | 0.998 ± 0.173 | 0.327 ± 0.14 | 0.316 ± 0.036 | 0.271 ± 0.0055 | 0.896 ± 0.57 | 0.655 ± 0.15 |
| L-Ala | 0.808 ± 0.18 | 0.660 ± 0.129 | 0.448 ± 0.065 | 0.957 ± 0.018 | 0.618 ± 0.17 | 0.905 ± 0.47 | 0.859 ± 0.014 | 1.26 ± 0.52 | 1.03 ± 0.087 |
| L-Ser | 0.213 ± 0.0046 | 0.0801 ± 0.0060 | 0.0744 ± 0.012 | 0.498 ± 0.10 | 0.186 ± 0.046 | 0.314 ± 0.17 | 0.170 ± 0.028 | 0.0849 ± 0.0079 | 0.0897 ± 0.023 |
| GABA | 0.00610 ± 0.0011 | 0.000253 ± 0.00036 | N.D. | 0.0740 ± 0.032 | 0.0335 ± 0.023 | 0.00264 ± 0.0019 | 0.00114 ± 0.0008 | N.D. | N.D. |
| Gly | 2.92 ± 0.033 | 1.55 ± 0.56 | 0.604 ± 0.20 | 4.91 ± 0.055 | 1.606 ± 0.17 | 1.33 ± 0.14 | 3.47 ± 0.42 | 7.82 ± 2.6 | 4.65 ± 1.4 |
| L-Glu | 0.307 ± 0.0047 | 0.110 ± 0.00024 | 1.82 ± 0.80 | 0.328 ± 0.074 | 0.0401 ± 0.057 | 0.428 ± 0.057 | 0.232 ± 0.058 | 0.180 ± 0.19 | 0.155 ± 0.052 |
| D-Pro | 0.00291 ± 0.0022 | N.D. | 0.00117 ± 0.0017 | N.D. | 0.00887 ± 0.0093 | N.D. | N.D. | N.D. | N.D. |
| L-Pro | 0.178 ± 0.013 | 0.0604 ± 0.0013 | 0.0467 ± 0.0031 | 0.214 ± 0.034 | 0.112 ± 0.017 | 0.210 ± 0.14 | 0.136 ± 0.0039 | 0.0524 ± 0.0044 | 0.0547 ± 0.0093 |
| L-Val | 0.169 ± 0.00066 | 0.0892 ± 0.0032 | 0.0684 ± 0.013 | 0.202 ± 0.039 | 0.141 ± 0.037 | 0.331 ± 0.18 | 0.173 ± 0.0094 | 0.0867 ± 0.028 | 0.109 ± 0.031 |
| L-Met | 0.183 ± 0.023 | 0.0215 ± 0.0020 | 0.0342 ± 0.012 | 0.203 ± 0.048 | 0.0427 ± 0.029 | 0.137 ± 0.081 | 0.0844 ± 0.0099 | 0.0522 ± 0.014 | 0.0418 ± 0.0014 |
| L-His | 0.0653 ± 0.013 | 0.0509 ± 0.0040 | 0.0541 ± 0.0087 | 0.0688 ± 0.0040 | 0.0468 ± 0.0036 | 0.110 ± 0.049 | 0.0658 ± 0.0033 | 0.0794 ± 0.0010 | 0.0539 ± 0.012 |
| D-Asp | 0.101 ± 0.091 | 0.0518 ± 0.0031 | 0.0332 ± 0.0080 | 0.140 ± 0.055 | 0.00477 ± 0.0067 | 0.0130 ± 0.00053 | N.D. | N.D. | 0.00609 ± 0.0019 |
| L-Asp | 0.558 ± 0.037 | 0.152 ± 0.053 | 0.237 ± 0.038 | 1.22 ± 0.25 | 0.349 ± 0.13 | 0.330 ± 0.20 | 0.411 ± 0.091 | 0.128 ± 0.0051 | 0.523 ± 0.11 |
| L-Ile | 0.158 ± 0.0046 | 0.0381 ± 0.00030 | 0.0405 ± 0.011 | 0.182 ± 0.039 | 0.0881 ± 0.027 | 0.236 ± 0.13 | 0.112 ± 0.011 | 0.0360 ± 0.017 | 0.0507 ± 0.015 |
| L-Leu | 0.368 ± 0.037 | 0.0664 ± 0.00037 | 0.0681 ± 0.0078 | 0.428 ± 0.096 | 0.157 ± 0.070 | 0.465 ± 0.28 | 0.225 ± 0.015 | 0.0593 ± 0.035 | 0.0765 ± 0.033 |
| L-Arg | 1.276 ± 0.097 | 0.158 ± 0.012 | 0.290 ± 0.14 | 1.59 ± 0.11 | 0.490 ± 0.075 | 0.605 ± 0.17 | 0.455 ± 0.052 | 0.483 ± 0.25 | 0.505 ± 0.15 |
| L-Trp | 0.0184 ± 0.00060 | 0.0197 ± 0.0014 | 0.0180 ± 0.0055 | 0.0175 ± 0.0010 | 0.0165 ± 0.0036 | 0.043 ± 0.02 | 0.0214 ± 0.0011 | 0.0247 ± 0.00093 | 0.0214 ± 0.0025 |
| L-Phe | 0.113 ± 0.0048 | 0.00330 ± 0.0045 | 0.00822 ± 0.0064 | 0.135 ± 0.020 | 0.0312 ± 0.044 | 0.190 ± 0.13 | 0.0866 ± 0.0025 | 0.0190 ± 0.0077 | 0.0236 ± 0.0048 |
| L-Orn | 0.00760 ± 0.011 | N.D. | N.D. | 0.00254 ± 0.0024 | 0.00713 ± 0.010 | N.D. | N.D. | N.D. | 0.00455 ± 0.0042 |
| L-Lys | 0.503 ± 0.075 | 0.0526 ± 0.026 | 0.0733 ± 0.041 | 0.403 ± 0.11 | 0.211 ± 0.050 | 0.390 ± 0.27 | 0.0325 ± 0.056 | 0.181 ± 0.080 | 0.233 ± 0.095 |
| L-Tyr | 0.0929 ± 0.042 | 0.0479 ± 0.0056 | 0.0806 ± 0.034 | 0.053 ± 0.0048 | 0.0986 ± 0.024 | 0.238 ± 0.10 | 0.132 ± 0.0049 | 0.0549 ± 0.0059 | 0.0832 ± 0.0099 |

**Table S3** Concentrations of amino acids in the tissues of kuruma prawn (mean ± SD [mmol/100 g-wet]).

| | Muscle | Hepatopancreas |
|---|---|---|
| L-Gln | 1.27 ± 0.080 | 1.15 ± 0.59 |
| β-Ala | N.D. | 0.0123 ± 0.012 |
| D-Ala | 0.499 ± 0.12 | 0.304 ± 0.14 |
| L-Ala | 0.598 ± 0.090 | 1.71 ± 0.73 |
| L-Ser | 0.0889 ± 0.0117 | 1.40 ± 0.59 |
| GABA | 0.000387 ± 0.00039 | 0.000585 ± 0.00053 |
| Gly | 12.3 ± 1.8 | 3.33 ± 2.2 |
| L-Glu | 1.26 ± 0.065 | 1.19 ± 0.56 |
| D-Pro | 0.00225 ± 0.0021 | N.D. |
| L-Pro | 3.69 ± 1.1 | 1.46 ± 0.55 |
| L-Val | 0.120 ± 0.043 | 1.80 ± 0.70 |
| L-Met | 0.0686 ± 0.015 | 0.713 ± 0.30 |
| L-His | 0.0831 ± 0.012 | 0.727 ± 0.33 |
| D-Asp | N.D. | 0.00655 ± 0.0034 |
| L-Asp | 0.114 ± 0.045 | 1.33 ± 0.57 |
| L-Ile | 0.0281 ± 0.013 | 1.18 ± 0.51 |
| L-Leu | 0.0650 ± 0.051 | 2.43 ± 1.1 |
| L-Arg | 3.95 ± 0.26 | 1.99 ± 0.85 |
| L-Trp | 0.0146 ± 0.0049 | 0.409 ± 0.19 |
| L-Phe | 0.00945 ± 0.016 | 0.983 ± 0.38 |
| L-Orn | 0.00564 ± 0.0035 | N.D. |
| L-Lys | 0.0965 ± 0.066 | 2.71 ± 0.96 |
| L-Tyr | 0.127 ± 0.037 | 0.887 ± 0.34 |

**Table S4** Results of similarity of percentages (SIMPER) analysis.

| Siphon | | | |
|---|---|---|---|
| Group 1 * Group 2 | Mean | | Cumulative contribution ratio (%) |
| | Group 1 | Group 2 | |
| Gly | 7.326 | 2.046 | 13.2 |
| L-Leu | 4.756 | 0.951 | 23.0 |
| L-Glu | 4.864 | 3.886 | 31.8 |
| L-Phe | 3.817 | 0.463 | 40.4 |
| L-Lys | 5.151 | 2.782 | 46.4 |
| D-Ala | 7.002 | 8.982 | 51.8 |
| L-Gln | 4.775 | 2.685 | 57.1 |
| L-Met | 4.472 | 2.590 | 61.8 |
| L-Ile | 4.236 | 2.425 | 66.5 |
| L-Ser | 5.279 | 3.593 | 70.7 |

| Foot | | | |
|---|---|---|---|
| Group 1 * Group 2 | Mean | | Cumulative contribution ratio (%) |
| | Group 1 | Group 2 | |
| L-Glu | 4.622 | 4.356 | 10.2 |
| L-Leu | 4.336 | 1.410 | 19.1 |
| L-Phe | 3.051 | 0.166 | 27.8 |
| D-Asp | 1.708 | 3.901 | 34.9 |
| β-Ala | 2.489 | 0.226 | 41.7 |
| L-Arg | 7.024 | 5.214 | 47.1 |
| Gly | 7.755 | 6.216 | 52.5 |
| L-Gln | 4.529 | 2.765 | 57.8 |
| L-Met | 4.064 | 2.461 | 62.6 |
| L-Ile | 3.778 | 2.187 | 67.4 |

| Adductor muscle | | | |
|---|---|---|---|
| Group 1 * Group 2 | Mean | | Cumulative contribution ratio (%) |
| | Group 1 | Group 2 | |
| L-Glu | 4.065 | 3.840 | 11.7 |
| L-Leu | 3.429 | 0.776 | 21.0 |
| Gly | 7.997 | 5.622 | 29.0 |
| L-Phe | 2.045 | 0.166 | 35.1 |
| D-Pro | 1.280 | 1.935 | 40.6 |
| L-Gln | 4.098 | 2.708 | 45.6 |
| L-Ile | 3.298 | 1.867 | 50.7 |
| D-Ala | 6.844 | 8.229 | 55.7 |
| L-Lys | 4.283 | 2.905 | 60.6 |
| L-Ser | 4.941 | 3.499 | 65.4 |

| Mantle | | | |
|---|---|---|---|
| Group 1 * Group 2 | Mean | | Cumulative contribution ratio (%) |
| | Group 1 | Group 2 | |
| L-Arg | 6.481 | 3.409 | 9.40 |
| L-Phe | 3.110 | 0.166 | 18.3 |
| Gly | 7.219 | 4.434 | 27.0 |
| L-Glu | 5.035 | 2.535 | 34.6 |
| L-Lys | 4.740 | 2.436 | 41.6 |
| L-Leu | 4.301 | 2.064 | 48.6 |
| L-Met | 4.014 | 2.382 | 53.5 |
| L-Gln | 4.664 | 3.153 | 58.1 |
| L-Ile | 3.836 | 2.520 | 62.3 |
| β-Ala | 1.652 | 1.297 | 66.4 |

| Mantle | | | |
|---|---|---|---|
| Group 1 * Group 3 | Mean | | Cumulative contribution ratio (%) |
| | Group 1 | Group 3 | |
| L-Arg | 6.481 | 0.133 | 12.3 |
| L-Glu | 5.035 | 0.388 | 21.3 |
| L-Lys | 4.740 | 0.138 | 30.2 |
| L-Leu | 4.301 | 0.215 | 38.1 |
| L-Met | 4.014 | 0.222 | 45.4 |
| L-Ile | 3.836 | 0.215 | 52.5 |
| Gly | 7.219 | 3.833 | 59.1 |
| L-Phe | 3.110 | 0.166 | 64.8 |
| L-Gln | 4.664 | 2.511 | 68.9 |
| L-Ser | 4.792 | 2.841 | 72.7 |

| Mantle | | | |
|---|---|---|---|
| Group 2 * Group 3 | Mean | | Cumulative contribution ratio (%) |
| | Group 2 | Group 3 | |
| L-Arg | 3.409 | 0.133 | 14.1 |
| L-Ile | 2.520 | 0.215 | 24.0 |
| L-Lys | 2.436 | 0.138 | 33.9 |
| L-Met | 2.382 | 0.222 | 43.2 |
| L-Glu | 2.535 | 0.388 | 52.4 |
| L-Leu | 2.064 | 0.215 | 60.4 |
| Gly | 4.434 | 3.833 | 68.2 |
| β-Ala | 1.297 | 0.226 | 72.8 |
| L-Ser | 3.834 | 2.841 | 77.0 |
| L-His | 3.139 | 2.264 | 80.8 |

| Gill | | | |
|---|---|---|---|
| Group 1 * Group 2 | Mean | | Cumulative contribution ratio (%) |
| | Group 1 | Group 2 | |
| L-Arg | 5.001 | 0.133 | 14.1 |
| L-Lys | 4.975 | 0.138 | 28.1 |
| L-Phe | 3.407 | 0.166 | 37.4 |
| L-Met | 3.972 | 1.086 | 45.7 |
| L-Glu | 4.442 | 6.919 | 53.3 |
| β-Ala | 1.947 | 0.226 | 58.0 |
| Gly | 6.650 | 5.893 | 62.1 |
| L-Leu | 4.849 | 3.461 | 66.0 |
| L-Tyr | 4.254 | 2.963 | 69.7 |
| D-Ala | 6.174 | 6.991 | 73.2 |

| Viscera | | | |
|---|---|---|---|
| Group 1 * Group 2 | Mean | | Cumulative contribution ratio (%) |
| | Group 1 | Group 2 | |
| L-Glu | 5.515 | 0.388 | 19.6 |
| β-Ala | 2.666 | 0.226 | 28.8 |
| L-Gln | 5.121 | 3.019 | 36.8 |
| GABA | 1.247 | 2.885 | 44.7 |
| D-Asp | 2.609 | 0.518 | 52.6 |
| L-Phe | 3.414 | 4.149 | 57.8 |
| L-Lys | 4.677 | 5.510 | 62.5 |
| D-Ala | 6.545 | 5.424 | 66.8 |
| D-Pro | 0.565 | 1.201 | 70.1 |
| Gly | 7.880 | 7.305 | 73.2 |
