# Supplementary material for: d-Alanine content in the marine edible bivalve Panopea japonica and evaluation of its associated enzyme activities
Source: Sci Rep. 2025 Jul 14;15:25415. doi: 10.1038/s41598-025-10379-2 (PMC12259925; doi:10.1038/s41598-025-10379-2)
Supplement: Supplementary file 3 — Supplementary Material 3 [file 41598_2025_10379_MOESM3_ESM.pdf]

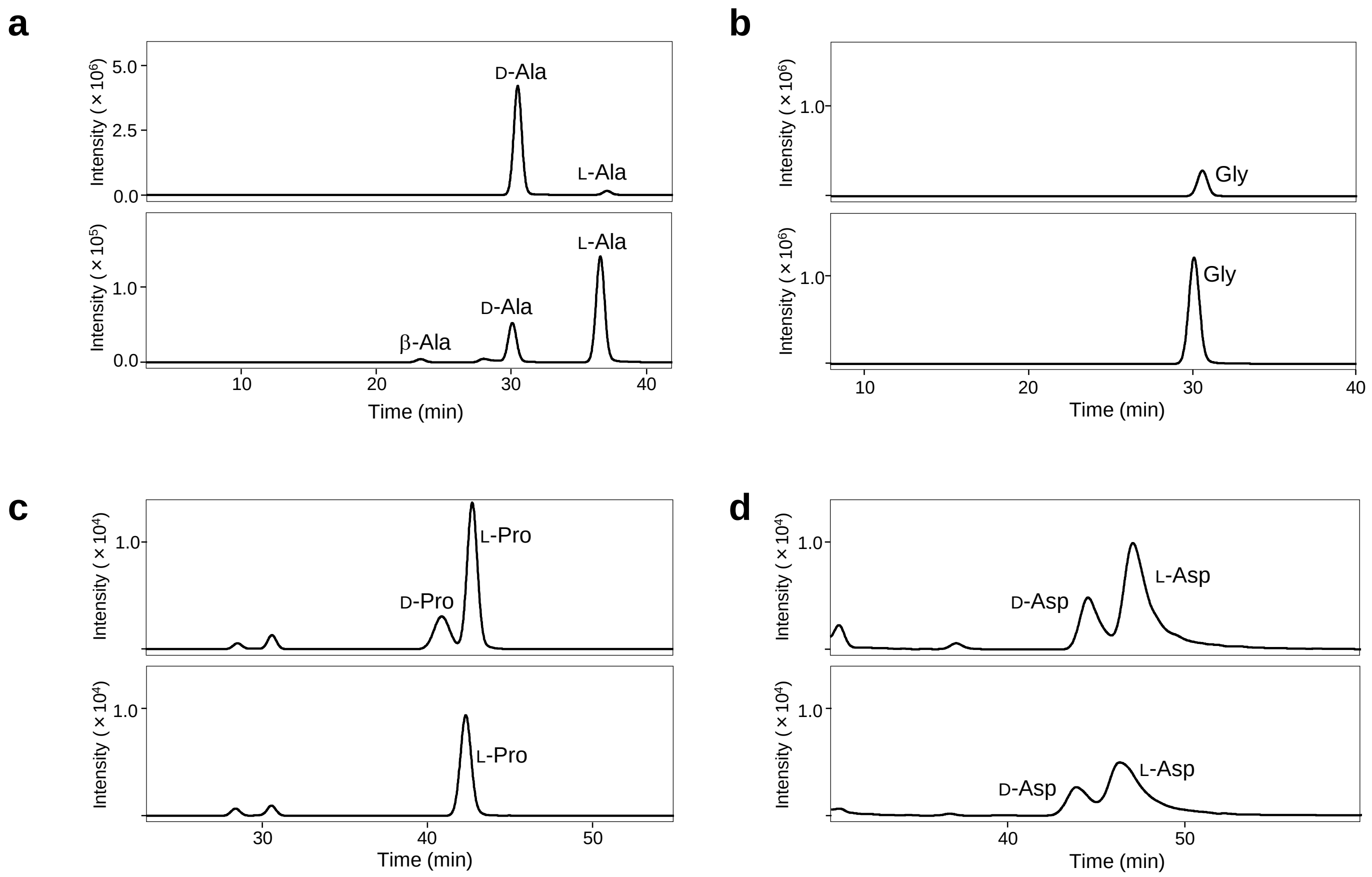

Fig. S1 Chromatograms of amino acids detected in *Panopea japonica* (upper panel) and *Ruditapes philippinarum* (lower panel). (a) Ala in the siphon, (b) Gly in the foot, (c) Pro in siphon, and (d) Asp in the gill.

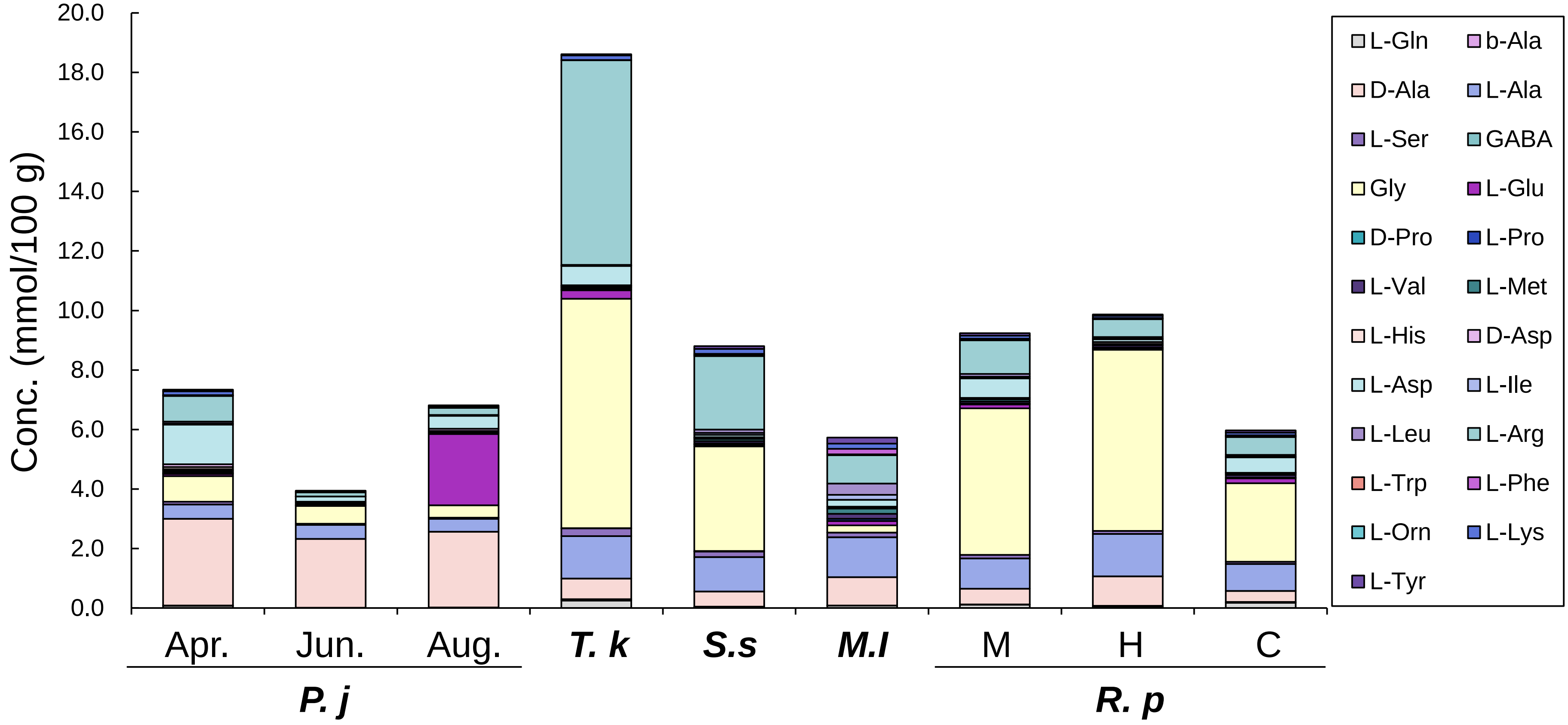

Fig. S2a Amino acids detected in the foot of bivalves (mmol/100 g-wet).
P. j: *Panopea japonica*; ***T. k***: *Tresus keenae*;
S. s: *Spisula sachalinensis*; ***M. l***: *Meretrix lusoria*;
R. p: *Ruditapes philippinarum*.
The M, H, and C on the ***R. p*** indicate their habitat,
i.e. Miyagi, Hokkaido, and Chiba prefectural areas.

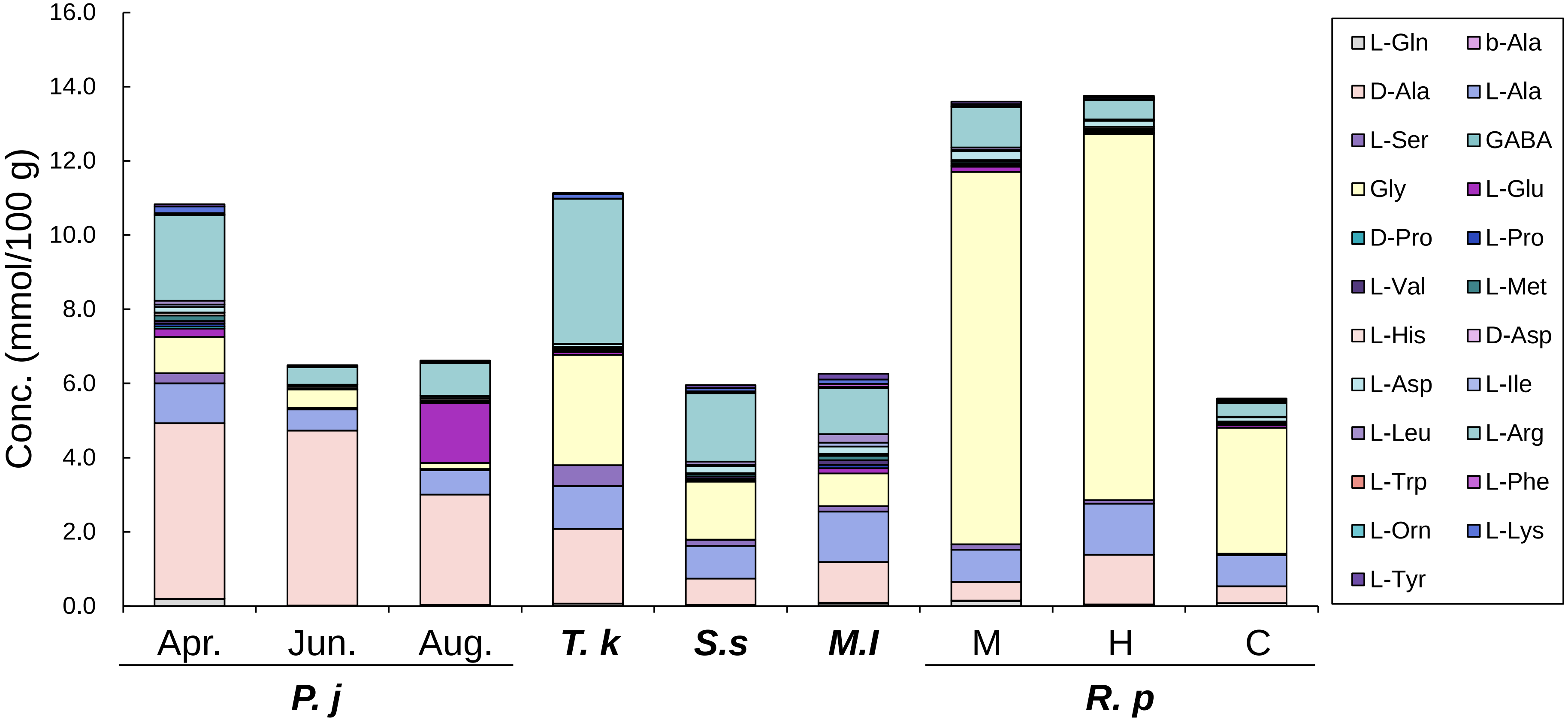

Fig. S2b Amino acids detected in the adductor muscle of bivalves (mmol/100 g-wet).
P. j: *Panopea japonica*; ***T. k***: *Tresus keenae*;
S. s: *Spisula sachalinensis*; ***M. l***: *Meretrix lusoria*;
R. p: *Ruditapes philippinarum*.
The M, H, and C on the ***R. p*** indicate their habitat,
i.e. Miyagi, Hokkaido, and Chiba prefectural areas.

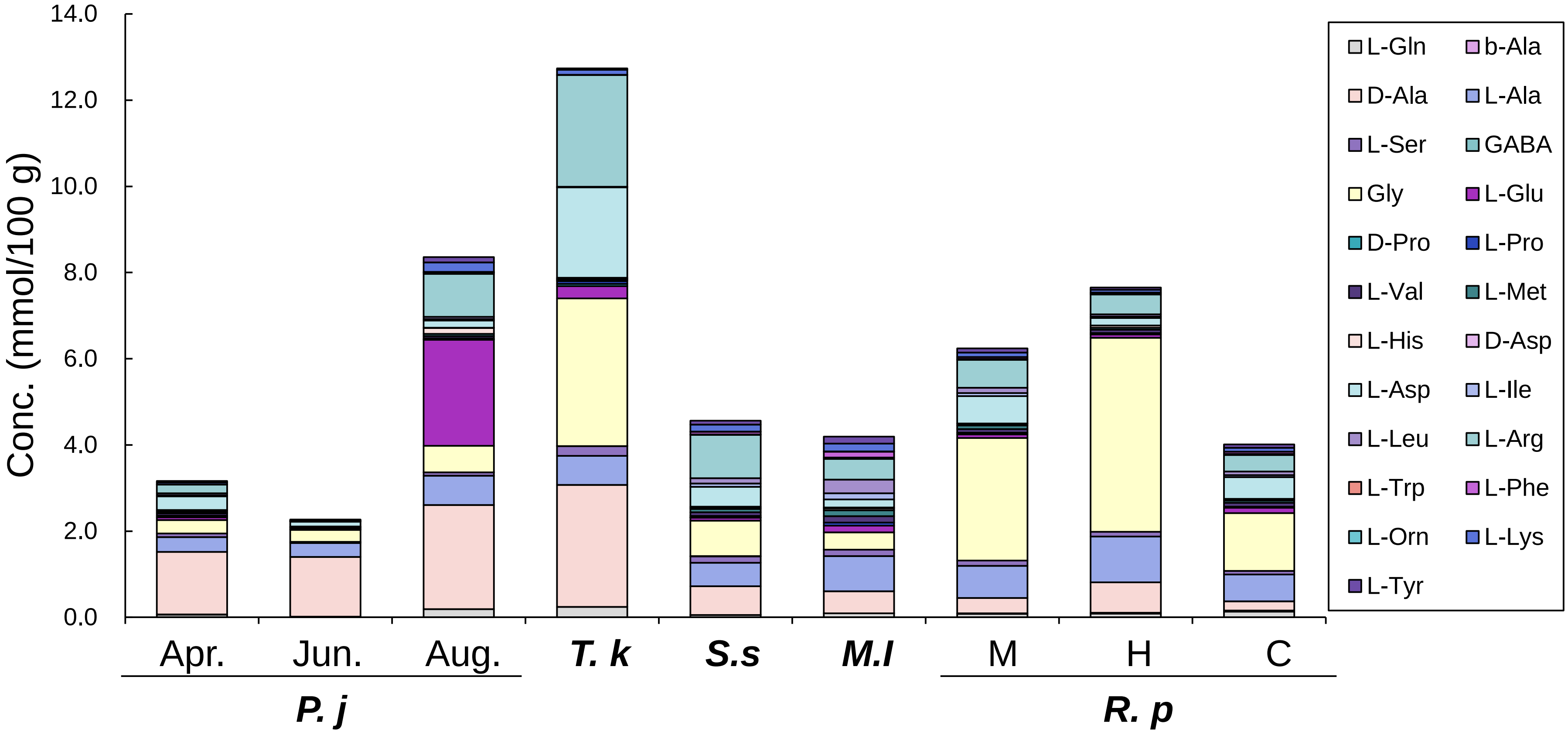

Fig. S2c Amino acids detected in the mantle of bivalves (mmol/100 g-wet).
P. j: *Panopea japonica*; ***T. k***: *Tresus keenae*;
S. s: *Spisula sachalinensis*; ***M. l***: *Meretrix lusoria*;
R. p: *Ruditapes philippinarum*.
The M, H, and C on the ***R. p*** indicate their habitat,
i.e. Miyagi, Hokkaido, and Chiba prefectural areas.

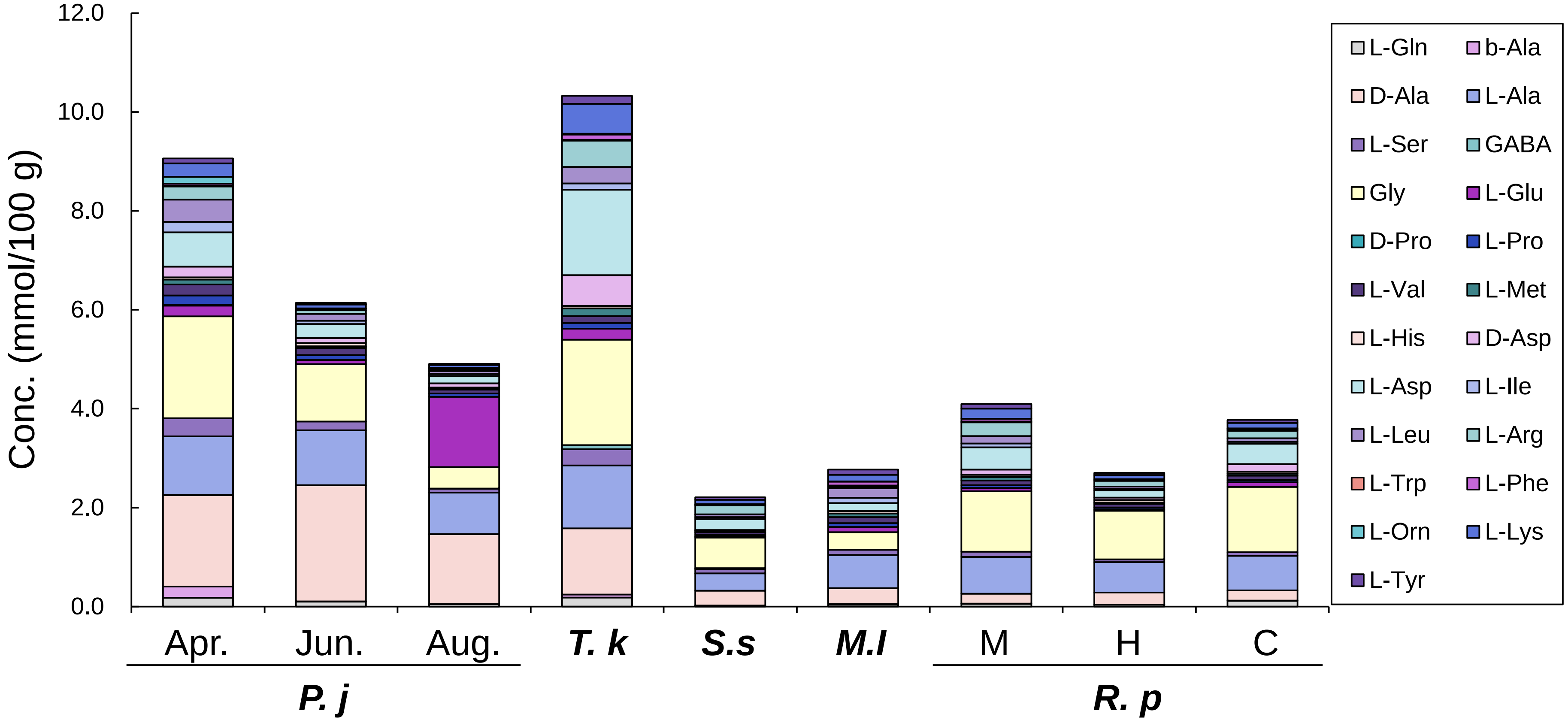

Fig. S2d Amino acids detected in the gill of bivalves (mmol/100 g-wet).
P. j: *Panopea japonica*; ***T. k***: *Tresus keenae*;
S. s: *Spisula sachalinensis*; ***M. l***: *Meretrix lusoria*;
R. p: *Ruditapes philippinarum*.
The M, H, and C on the ***R. p*** indicate their habitat,
i.e. Miyagi, Hokkaido, and Chiba prefectural areas.

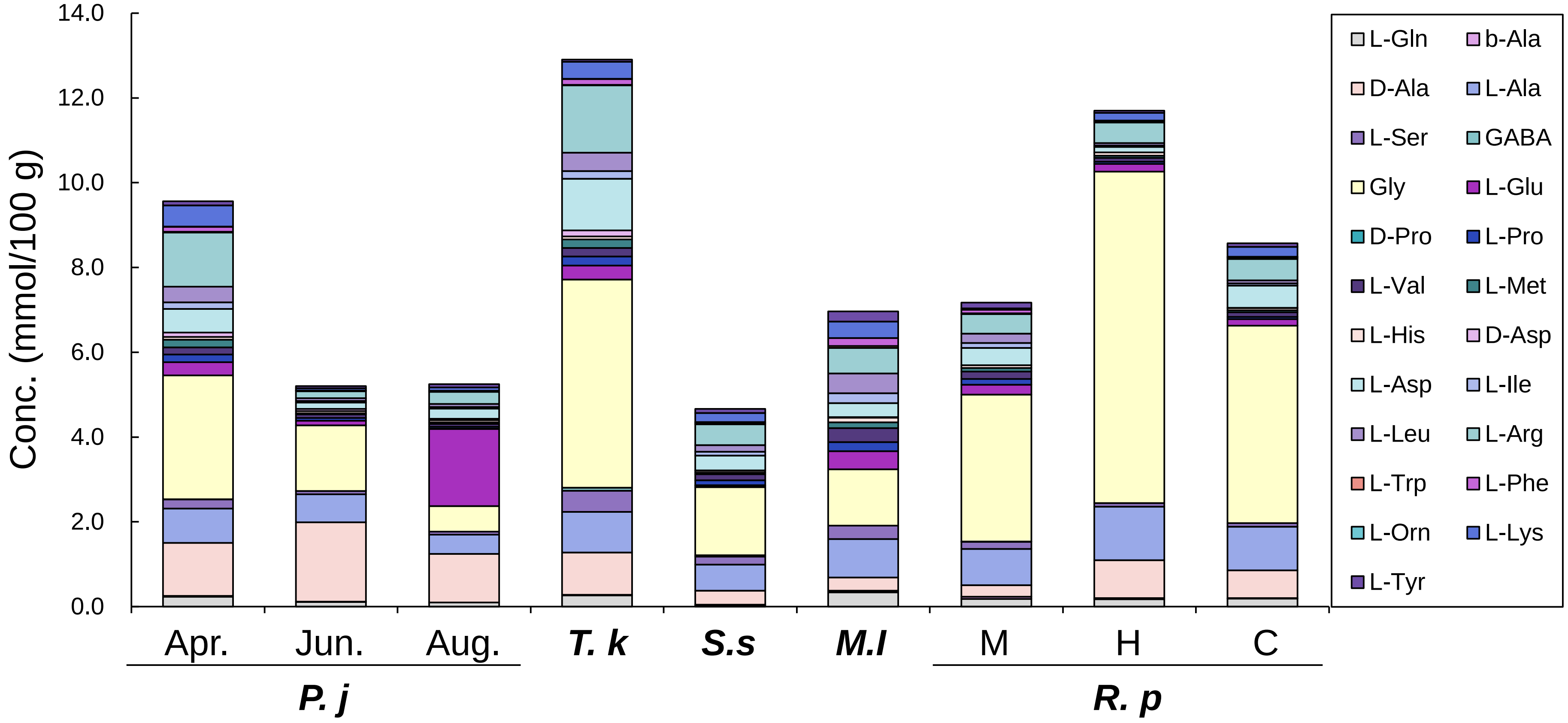

Fig. S2e Amino acids detected in the viscera of bivalves (mmol/100 g-wet).
P. j: *Panopea japonica*; ***T. k***: *Tresus keenae*;
S. s: *Spisula sachalinensis*; ***M. l***: *Meretrix lusoria*;
R. p: *Ruditapes philippinarum*.
The M, H, and C on the ***R. p*** indicate their habitat,
i.e. Miyagi, Hokkaido, and Chiba prefectural areas.

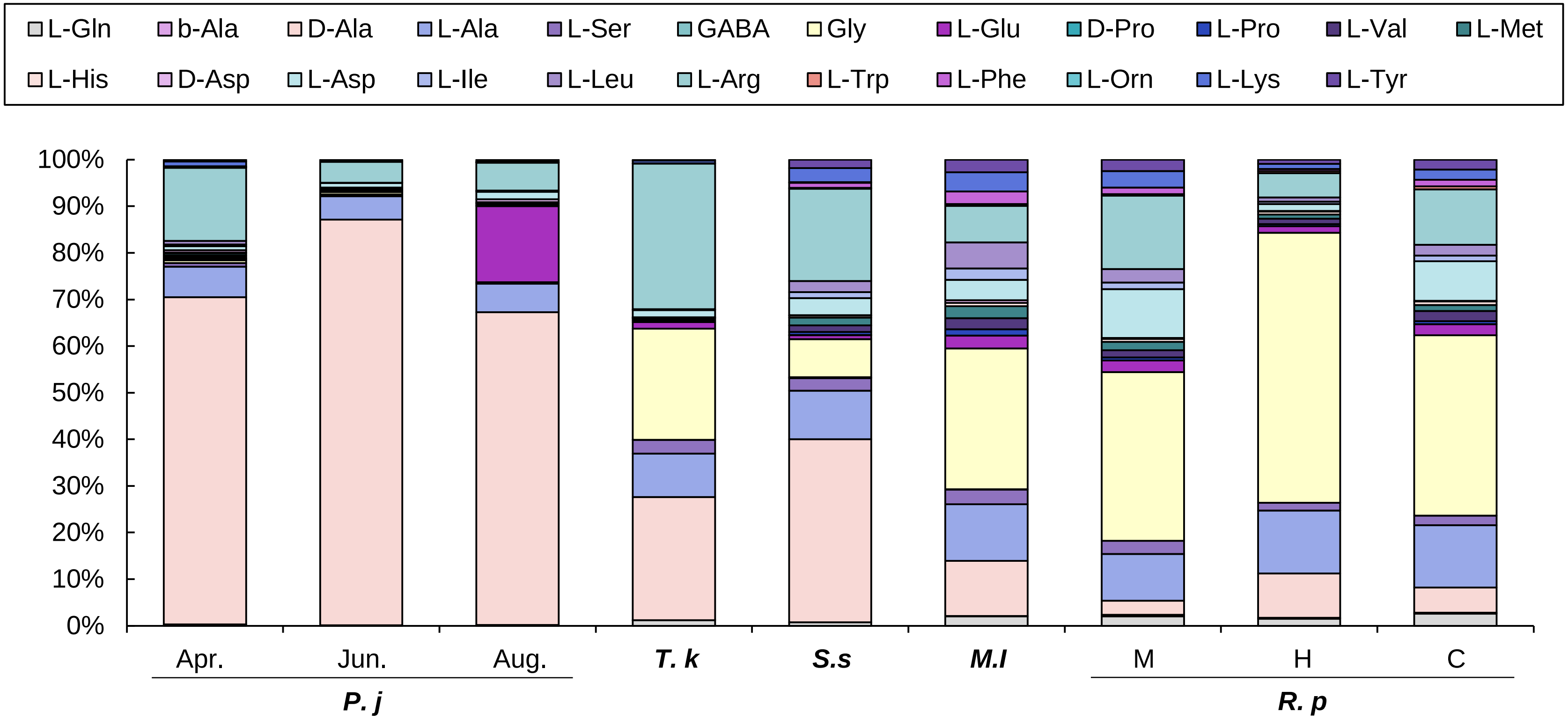

Fig. S3a Amino acid compositions (%) in the siphon of bivalves.
P. j: *Panopea japonica*; ***T. k***: *Tresus keenae*;
S. s: *Spisula sachalinensis*; ***M. l***: *Meretrix lusoria*;
R. p: *Ruditapes philippinarum*.
The M, H, and C on the ***R. p*** indicate their habitat,
i.e. Miyagi, Hokkaido, and Chiba prefectural areas.

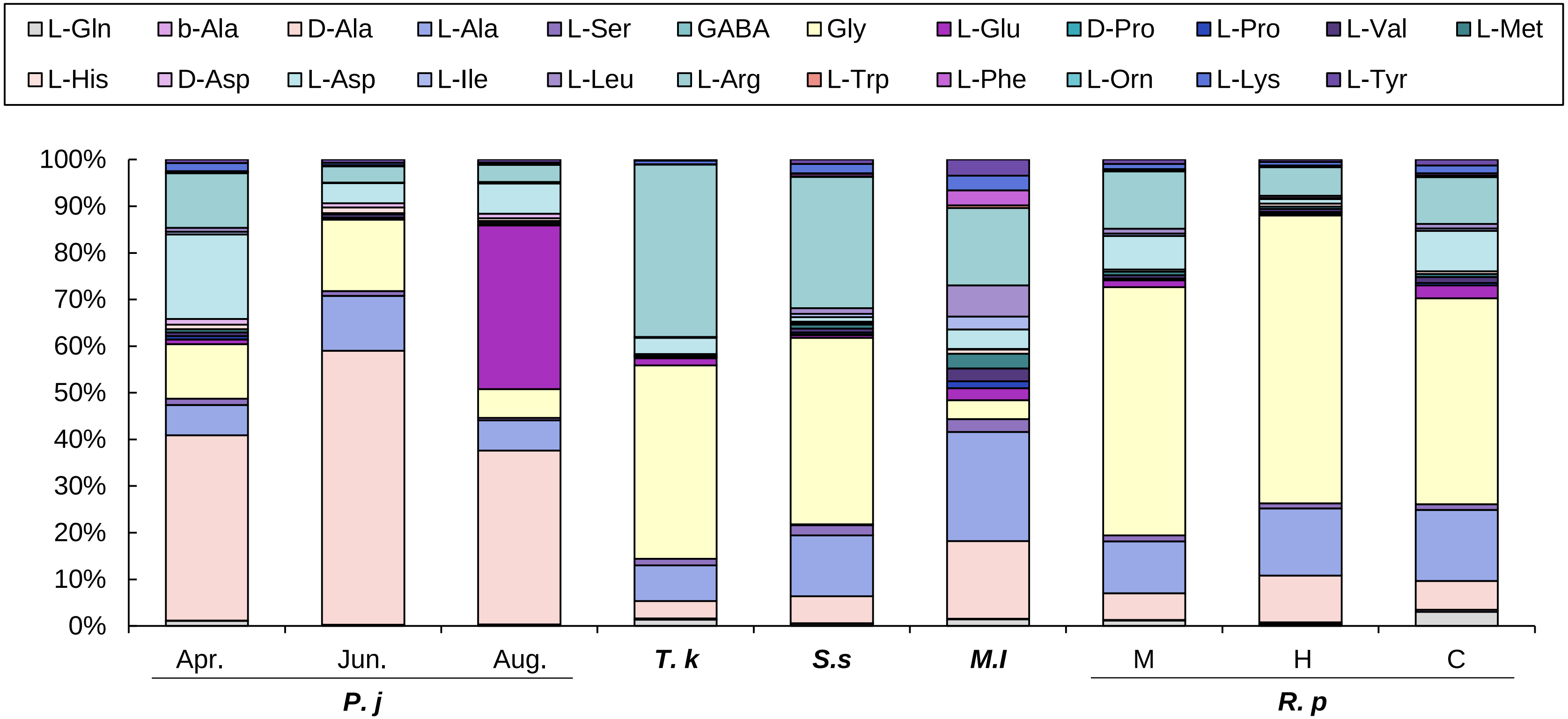

Fig. S3b Amino acid compositions (%) in the foot of bivalves.
P. j: *Panopea japonica*; ***T. k***: *Tresus keenae*;
S. s: *Spisula sachalinensis*; ***M. l***: *Meretrix lusoria*;
R. p: *Ruditapes philippinarum*.
The M, H, and C on the ***R. p*** indicate their habitat,
i.e. Miyagi, Hokkaido, and Chiba prefectural areas.

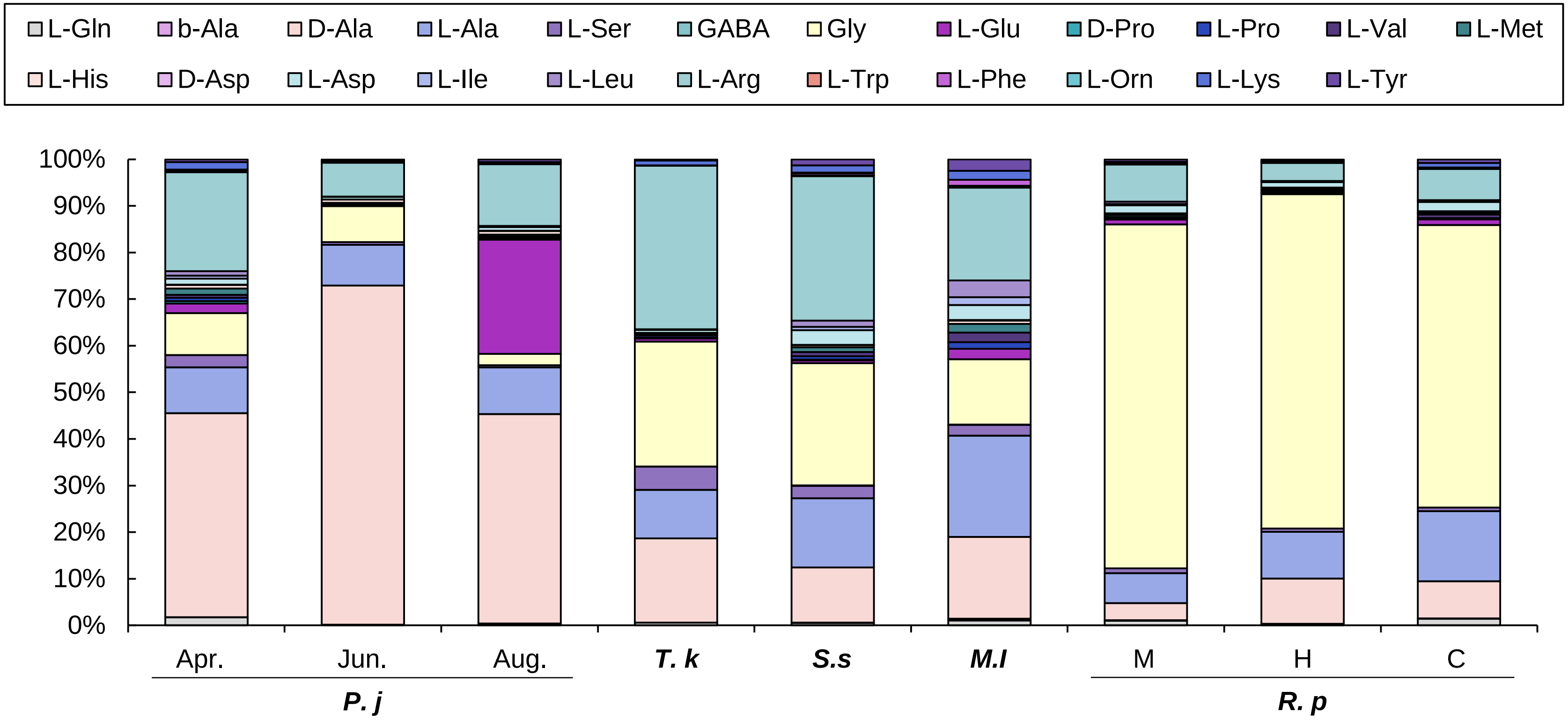

Fig. S3c Amino acid compositions (%) in the adductor muscle of bivalves.
P. j: *Panopea japonica*; ***T. k***: *Tresus keenae*;
S. s: *Spisula sachalinensis*; ***M. l***: *Meretrix lusoria*;
R. p: *Ruditapes philippinarum*.
The M, H, and C on the ***R. p*** indicate their habitat,
i.e. Miyagi, Hokkaido, and Chiba prefectural areas.

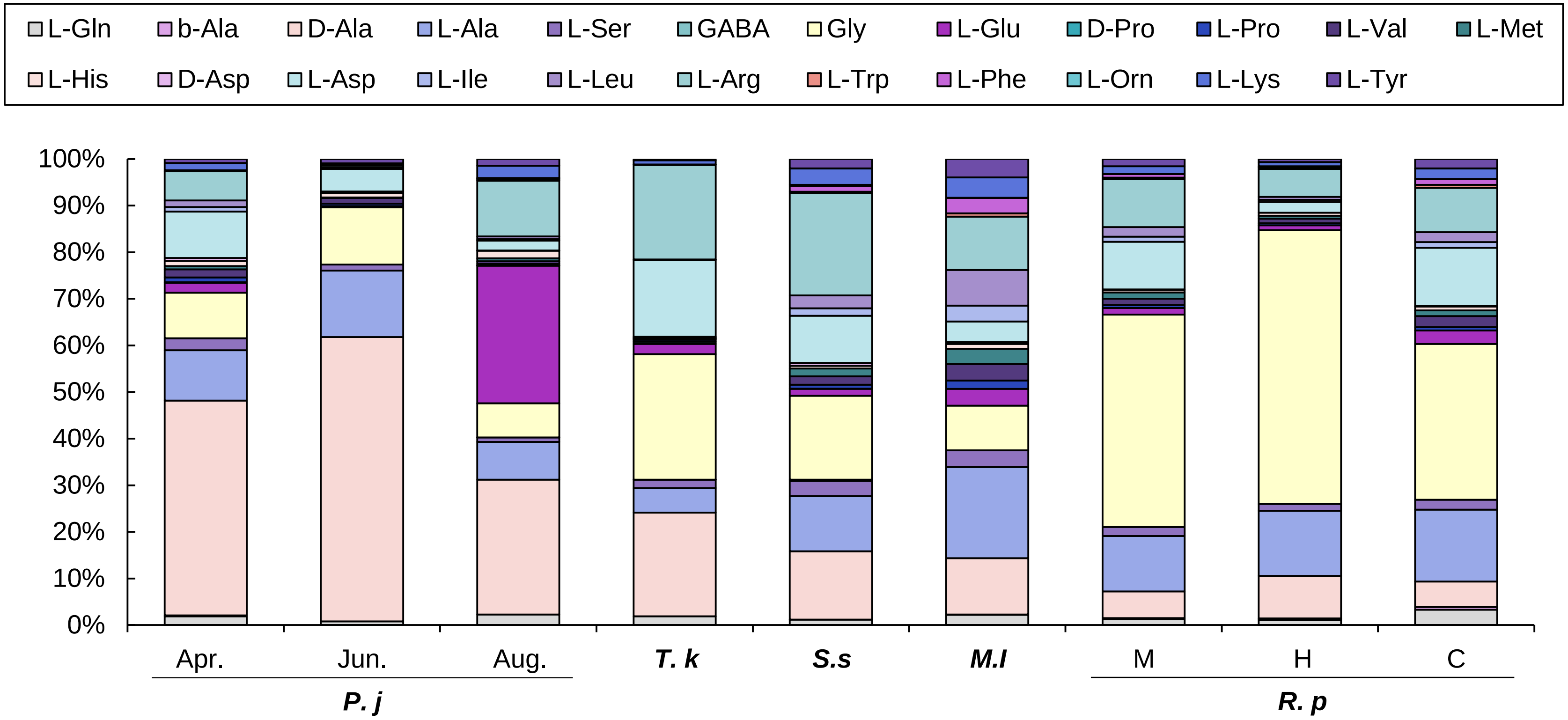

Fig. S3d Amino acid compositions (%) in the mantle of bivalves.
P. j: *Panopea japonica*; ***T. k***: *Tresus keenae*;
S. s: *Spisula sachalinensis*; ***M. l***: *Meretrix lusoria*;
R. p: *Ruditapes philippinarum*.
The M, H, and C on the ***R. p*** indicate their habitat,
i.e. Miyagi, Hokkaido, and Chiba prefectural areas.

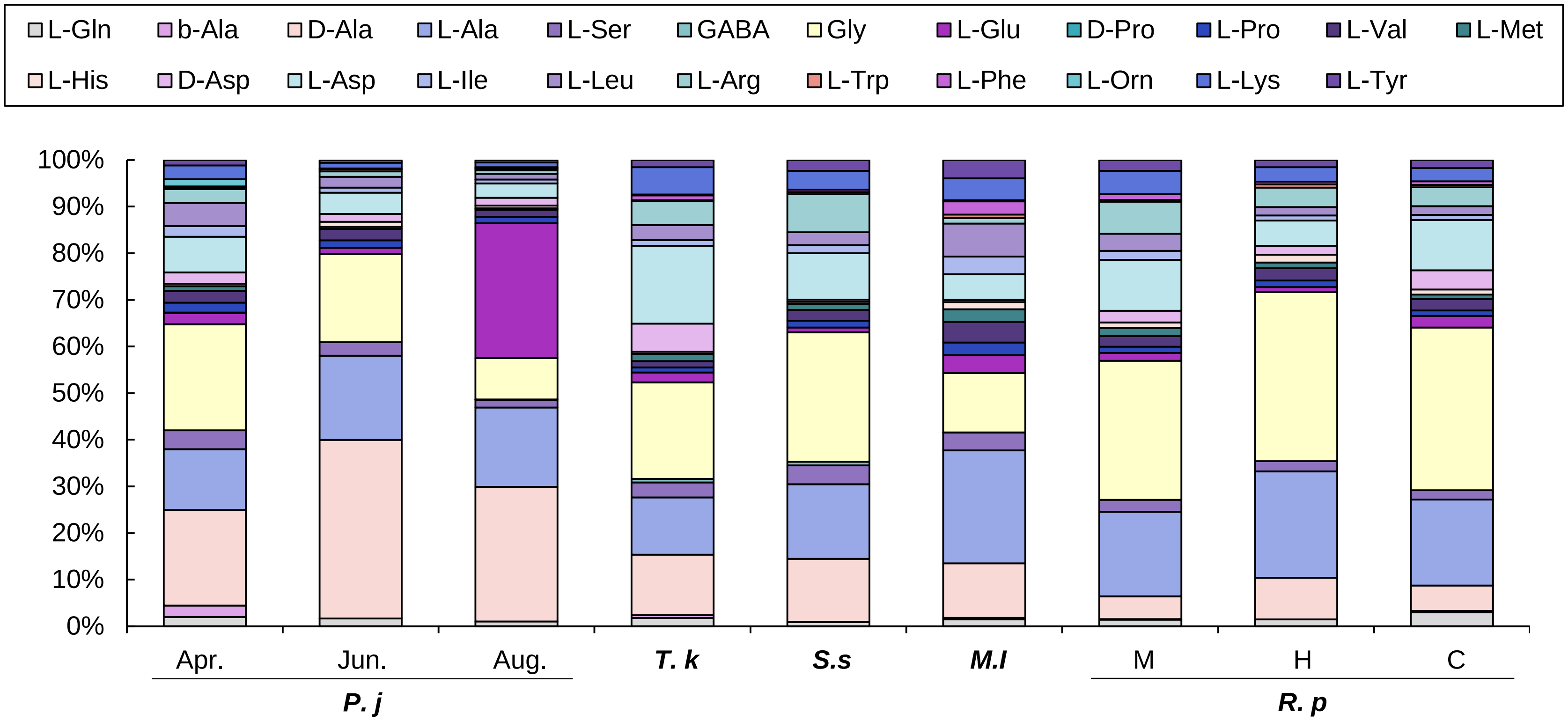

Fig. S3e Amino acid compositions (%) in the gill of bivalves.
P. j: *Panopea japonica*; ***T. k***: *Tresus keenae*;
S. s: *Spisula sachalinensis*; ***M. l***: *Meretrix lusoria*;
R. p: *Ruditapes philippinarum*.
The M, H, and C on the ***R. p*** indicate their habitat,
i.e. Miyagi, Hokkaido, and Chiba prefectural areas.

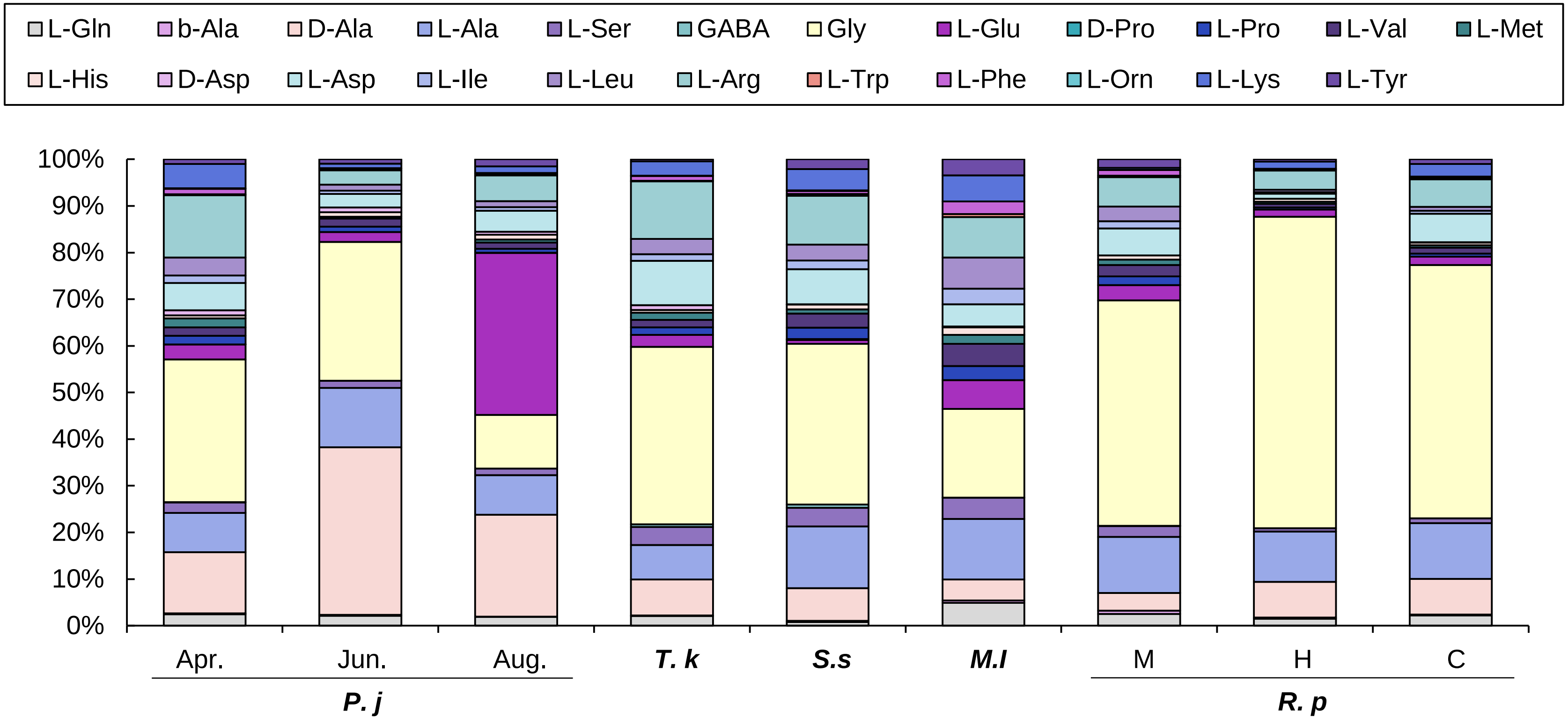

Fig. S3f Amino acid compositions (%) in the viscera of bivalves.
P. j: *Panopea japonica*; ***T. k***: *Tresus keenae*;
S. s: *Spisula sachalinensis*; ***M. l***: *Meretrix lusoria*;
R. p: *Ruditapes philippinarum*.
The M, H, and C on the ***R. p*** indicate their habitat,
i.e. Miyagi, Hokkaido, and Chiba prefectural areas.

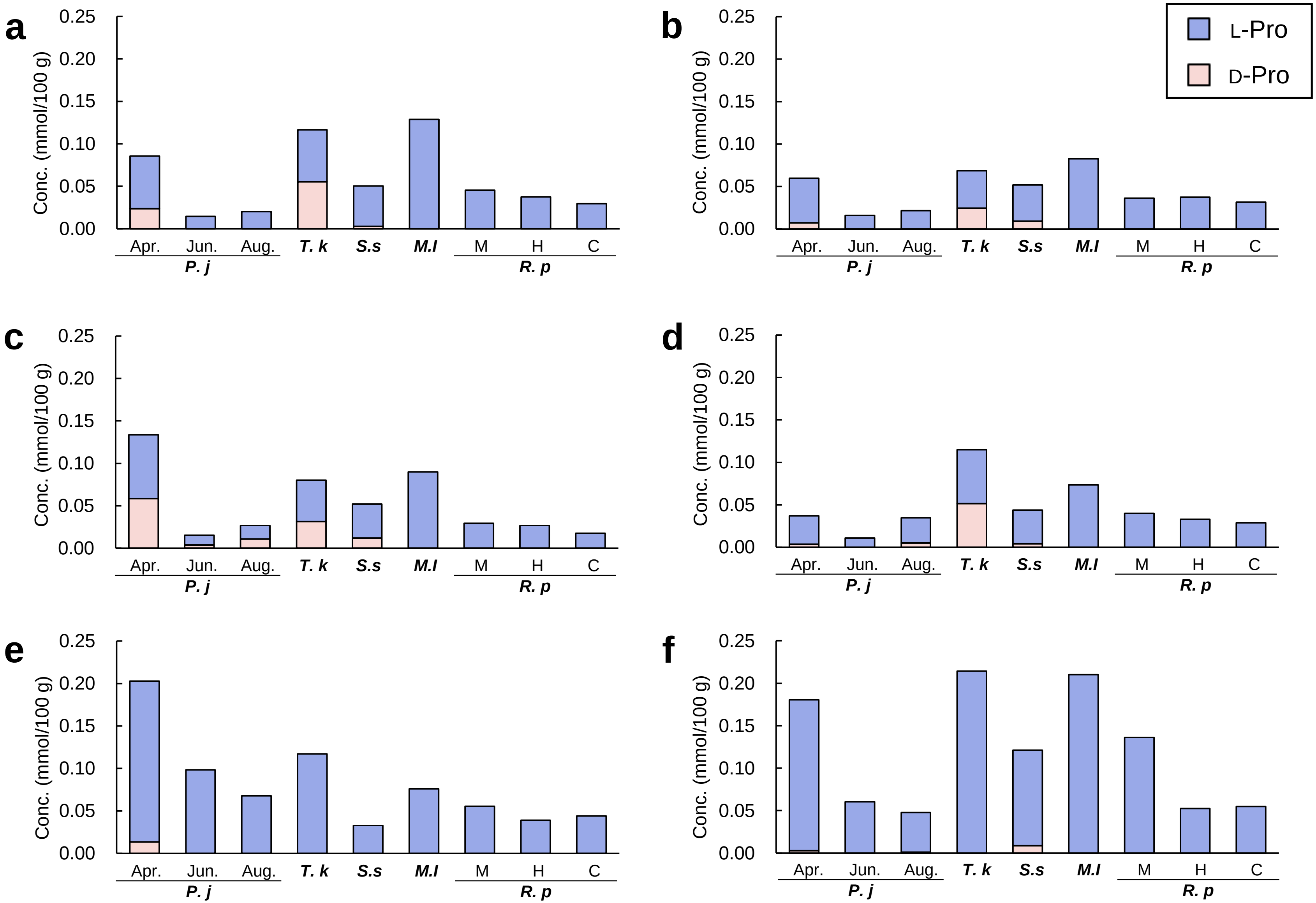

Fig. S4 D-Pro and L-Pro concentrations detected in various tissues of bivalves (mmol/100 g-wet).
(a) siphon, (b) foot, (c) adductor muscle, (d) mantle, (e) gill, and (f) viscera.
P. j: *Panopea japonica*; ***T. k***: *Tresus keenae*; ***S. s***: *Spisula sachalinensis*;
M. l: *Meretrix lusoria*; ***R. p***: *Ruditapes philippinarum*.
The M, H, and C on the ***R. p*** indicate their habitat, *i.e.* Miyagi, Hokkaido, and Chiba prefectural areas.

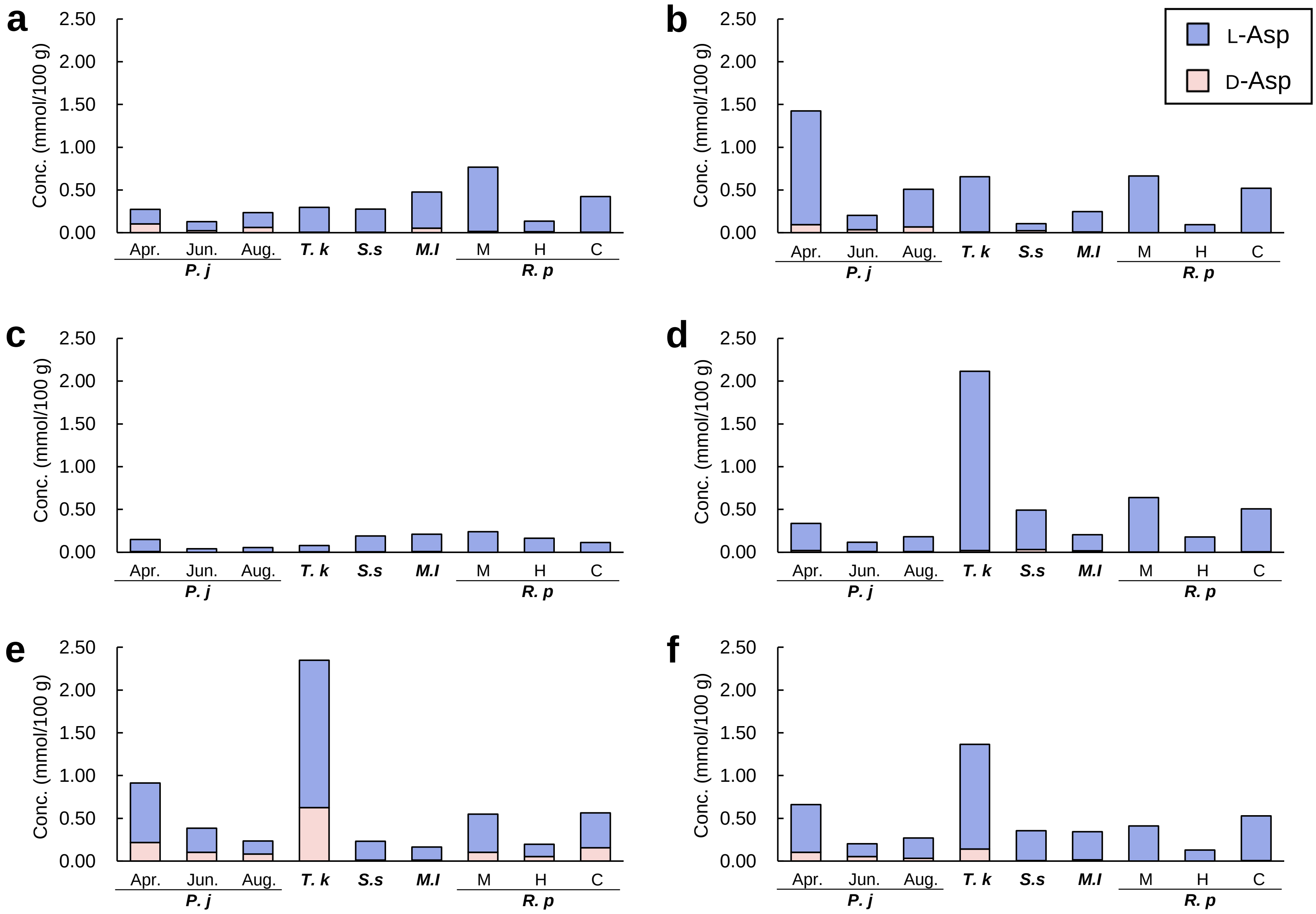

Fig. S5 D-Asp and L-Asp concentrations detected in various tissues of bivalves (mmol/100 g-wet).
(a) siphon, (b) foot, (c) adductor muscle, (d) mantle, (e) gill, and (f) viscera.
P. j: *Panopea japonica*; ***T. k***: *Tresus keenae*; ***S. s***: *Spisula sachalinensis*;
M. l: *Meretrix lusoria*; ***R. p***: *Ruditapes philippinarum*.
The M, H, and C on the ***R. p*** indicate their habitat, *i.e.* Miyagi, Hokkaido, and Chiba prefectural areas.

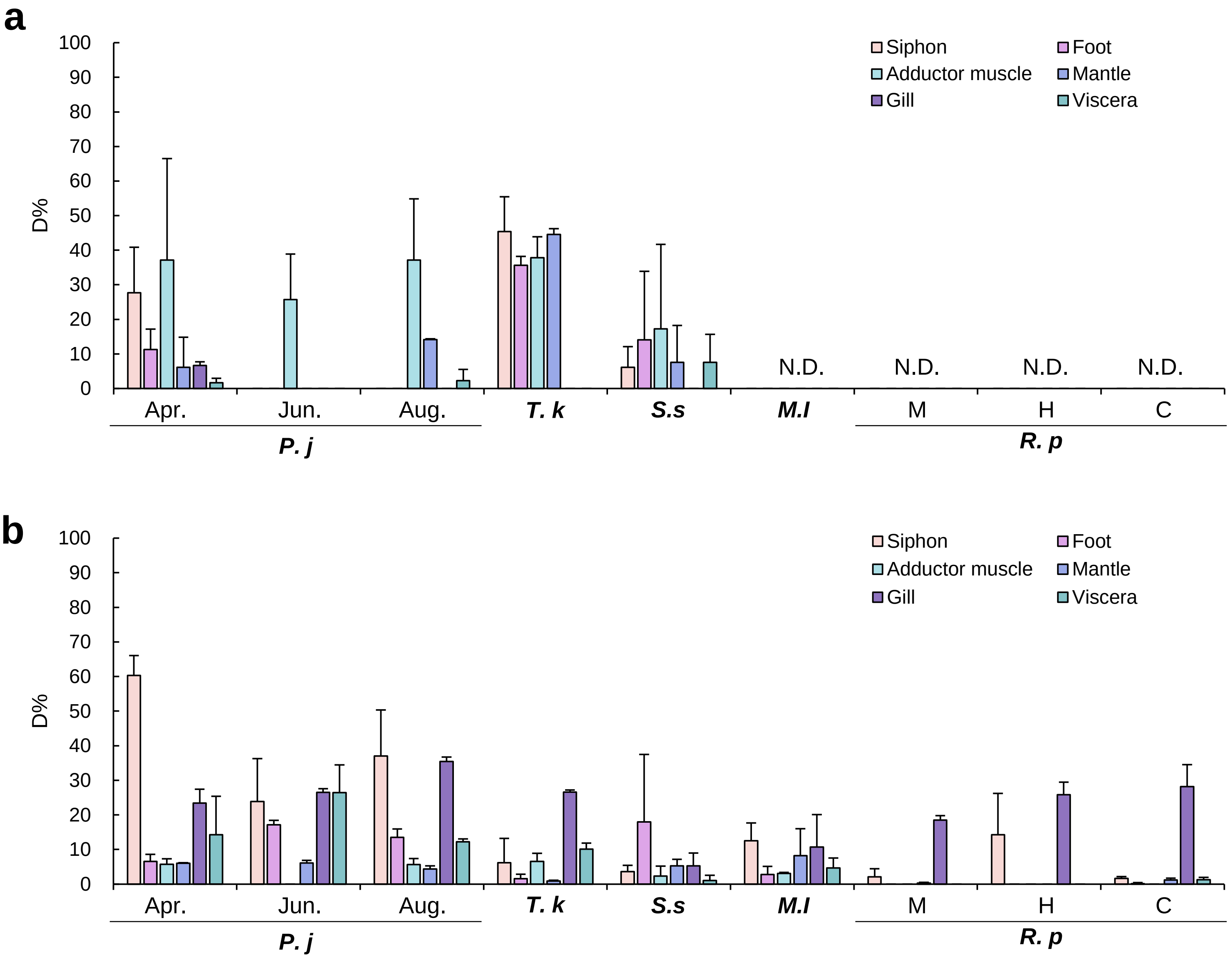

Fig. S6 The percentage of D-Pro (a) and D-Asp (b) to total Pro and Asp detected in each tissue of bivalves.
P. j: *Panopea japonica*; ***T. k***: *Tresus keenae*; ***S. s***: *Spisula sachalinensis*;
M. l: *Meretrix lusoria*; ***R. p***: *Ruditapes philippinarum*.
The M, H, and C on the ***R. p*** indicate their habitat, *i.e.* Miyagi, Hokkaido, and Chiba prefectural areas.

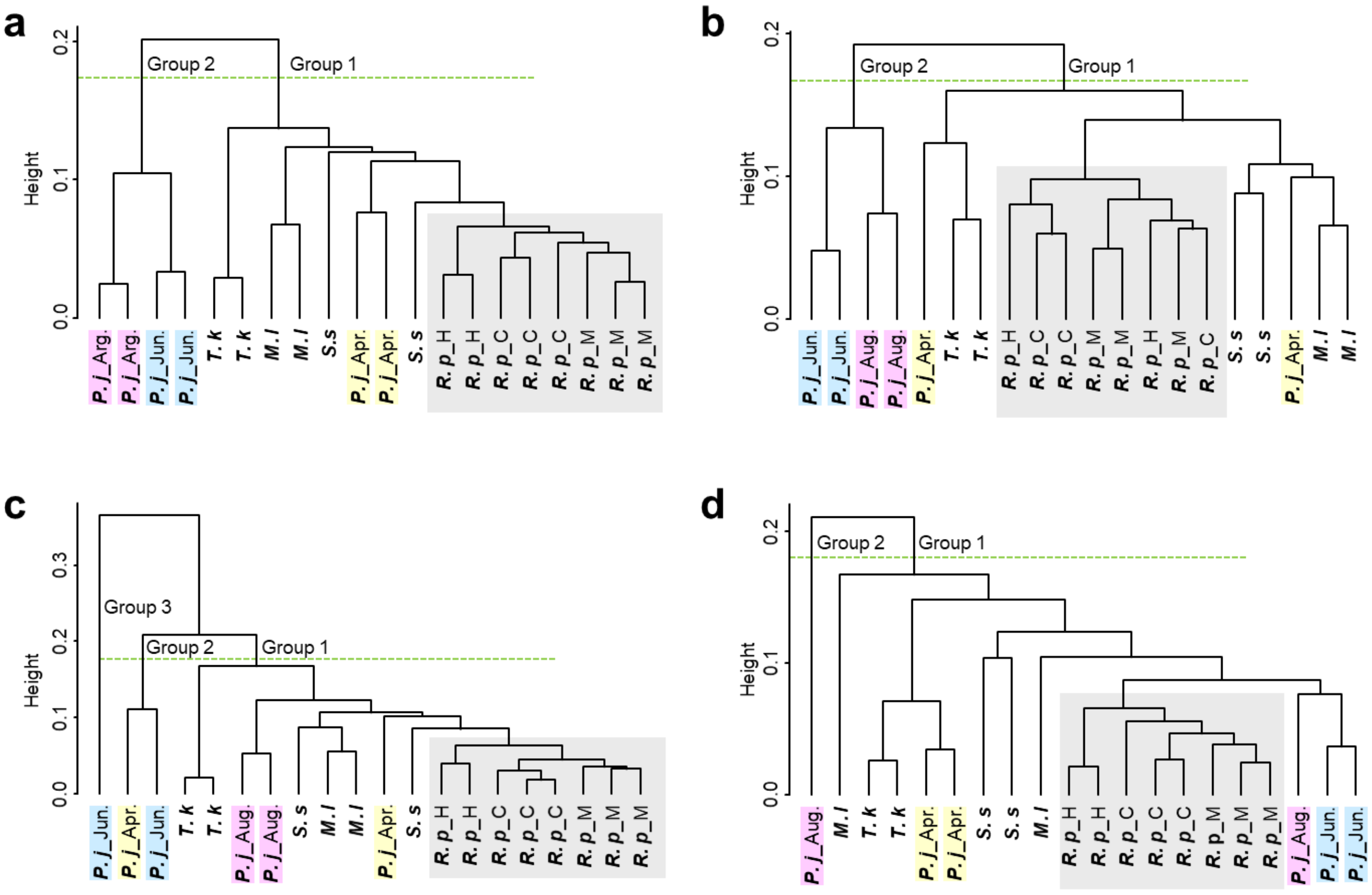

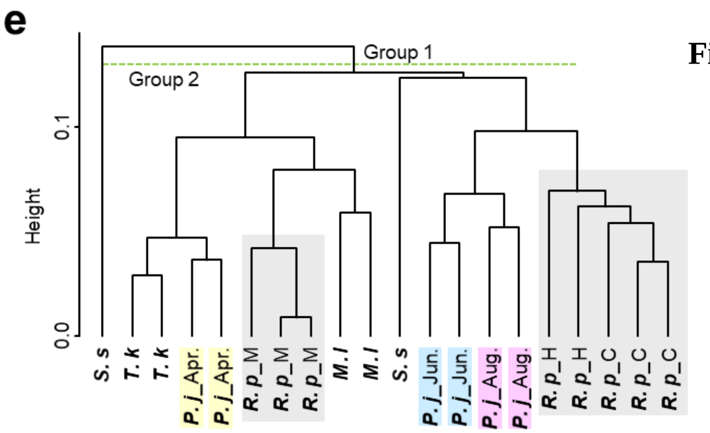

Fig. S7 Cluster analysis based on the concentrations of amino acids detected in the tissue of bivalves. Marked in light gray are ***R. p***. The ***P. j*** in April, June and August were marked in yellow, light blue and pink, respectively.

(a) foot, (b) adductor muscle, (c) mantle, (d) gill, and (e) viscera.

P. j: *Panopea japonica*; ***T. k***: *Tresus keenae*;
S. s: *Spisula sachalinensis*; ***M. l***: *Meretrix lusoria*;
R. p: *Ruditapes philippinarum*.

The M, H, and C on the ***R. p*** indicate their habitat, *i.e.* Miyagi, Hokkaido, and Chiba prefectural areas.
